# Short and long-term outcomes of percutaneous coronary intervention in patients with active or prior history of cancer: a systematic review and *meta*-analysis

**DOI:** 10.1016/j.ijcha.2025.101806

**Published:** 2025-09-22

**Authors:** Nikolaos Vythoulkas-Biotis, David-Dimitris Chlorogiannis, Theoni Theodoropoulou, Ioannis Gialamas, Evangelos Oikonomou, Konstantinos Kalogeras, Helena Michalopoulou, Gerasimos Siasos, Manolis Vavuranakis

**Affiliations:** a Third Department of Cardiology, “Sotiria” Chest Diseases Hospital, School of Medicine, National and Kapodistrian University of Athens, Athens, Greece; b Department of Radiology, Brigham and Women’s Hospital, Harvard Medical School, MA, USA; c First Department of Cardiology, Hippokration General Hospital, School of Medicine, National and Kapodistrian University of Athens, Athens, Greece

**Keywords:** Percutaneous coronary intervention, Coronary artery disease, Cancer, Outcomes, Meta-analysis

## Abstract

**Background:**

Percutaneous coronary intervention (PCI) in patients with active or prior history of cancer presents a challenge due to the increased rates of cardiovascular complications. Therefore, we aimed to evaluate the mortality rates, in addition to early and long-term adverse cardiovascular outcomes in this high-risk population.

**Methods:**

A systematic literature search was conducted across PubMed, Cochrane, and Scopus databases to identify eligible studies comparing clinical outcomes between patients with active or prior history of cancer and patients without cancer. Our primary outcomes were all-cause mortality and cardiovascular mortality at different time points. Secondary outcomes included bleeding, stroke, recurrent myocardial infarction (MI), and heart failure events between the two groups.

**Results:**

Overall, 18 studies with a total of 8 million patients were included. Active or prior history of cancer was associated with increased in-hospital all-cause mortality (RR: 1.43; 95 % CI: 1.03–1.99; p = 0.03), 1-year all-cause mortality (RR: 2.35; 95 % CI: 1.75–3.16; p < 0.001), as well as, increased 1-year cardiovascular mortality (RR: 1.35; 95 % CI: 1.15–1.59; p < 0.001) compared to patients without a history of cancer. Patients with active or prior history of cancer have higher rates of in-hospital (RR: 1.77; 95 % CI: 1.75–1.79; p < 0.001), 1-year (RR: 1.63; 95 %CI: 1.26–2.11; p < 0.001), and long-term bleeding events (RR: 2.08; 95 % CI: 1.30–3.35; p < 0.003) compared to patients without cancer. No significant differences were recorded regarding recurrent MI between the two groups.

**Conclusions:**

Active or prior history of cancer was associated with a negative impact on early and long-term clinical outcomes in patients undergoing PCI. These findings underline the importance of individualized and multidisciplinary approaches when treating this high-risk population.

## Highlights

• Cancer patients experience higher rates of all-cause and cardiovascular mortality after PCI.
• Cancer patients demonstrate higher rates of bleeding, stroke, and heart failure after PCI.
• Need for multidisciplinary approaches when managing cancer patients undergoing PCI.
• Correct balance between thrombotic and bleeding risk can be challenging.

## Graphical abstract

The present illustration depicts the impact of active or prior history of cancer on clinical outcomes in patients undergoing percutaneous coronary intervention. The findings of this systematic review and *meta*-analysis of 18 studies encompassing more than 8 million patients suggest that cancer comorbidity is associated with higher mortality rates, in addition to major cardiovascular events in different time points. Notably, there was no difference in recurrent myocardial infarction rates between the two groups across all time points.

**Figure:**
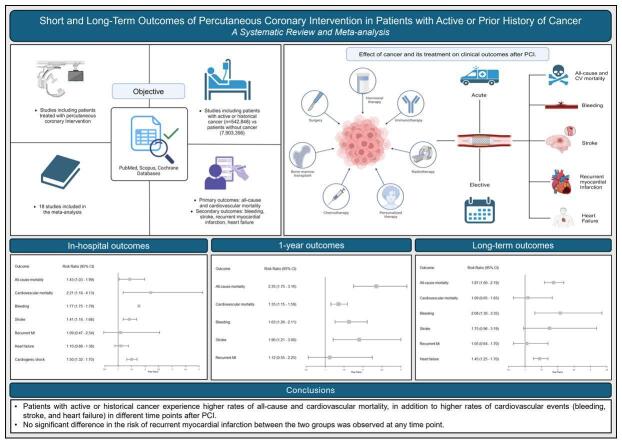

## 1 Introduction

Cancer and cardiovascular disease represent the predominant causes of morbidity and mortality on a global scale [1,2]. In 2024, 2 million new cancer cases and 611,720 cancer deaths are estimated to occur in the United States. From 1991 to 2021, cancer mortality continued to decline preventing over 4 million deaths [3]. The increased incidence of cancer diagnosis and the decline in cancer mortality have resulted in a higher prevalence of cardiovascular diseases among these patients, particularly coronary artery disease (CAD).[4] Common risk factors, including obesity, tobacco use, and advanced age precipitate the coexistence of both cancer and CAD.[[5], [6], [7]] In addition, it has been established that many cancer treatments, including chemotherapy, radiotherapy, and immunotherapy, can increase the likelihood of developing cardiovascular diseases by accelerating atherosclerosis and CAD [[8], [9], [10], [11]].

Percutaneous coronary intervention (PCI) is the most common method of coronary revascularization, indicated even in patients with multiple comorbidities [12,13] and extensive CAD [14,15]. Patients with active or prior history of cancer face both ischemic (stent thrombosis or acute myocardial infarction (AMI)) and bleeding complications following PCI, which compete in clinical decision making [16,17]. Several observational studies demonstrated that cancer patients face higher risk of both major adverse cardiovascular events (MACE) and bleeding complications [18,19]. Cancer patients are especially prone to these complications because malignancy promotes a hypercoagulable or prothrombotic state due to increased platelet activation and aggregation [20]. Additionally, chemotherapy can induce thrombocytopenia in cancer patients, increasing the risk of bleeding complications [21,22]. Therefore, patients with history or active cancer are usually excluded from randomized clinical trials [23,24] of the cardiovascular domain and most of the clinical outcome data following PCI in cancer patients are derived from observational or single-center studies, as demonstrated by previous *meta*-analyses [[25], [26], [27]]. While the short-term complications regarding cancer patients following PCI have been previously demonstrated, evidence on long-term outcomes remain unclear.

The objective of the present systematic review and *meta*-analysis was to synthesize contemporary evidence on the impact of active or prior history of cancer on short and long-term outcomes following PCI, focusing on mortality rates and cardiovascular events.

## 2 Materials and methods

This systematic review and *meta*-analysis was conducted according to the Preferred Reporting Items for Systematic-Review and Meta-Analysis (PRISMA) [28]. The study protocol has been prospectively registered in the International Prospective Register of Systematic Reviews (PROSPERO, ID: CRD42024570123).

### 2.1 Search strategy

A systematic literature searching PubMed, Scopus, and Cochrane databases was conducted from inception until July 17 of 2024. The reference lists of the included studies and prior *meta*-analyses were also screened to identify additional eligible studies. A more comprehensive presentation of the search strategy and the keywords used is presented in Supplemental Table 1.

**Table 1:** Characteristics of the studies included in the systematic review and *meta*-analysis.

Study	Year	Country	Study design	Total Sample Size Cancer	Total Sample Size no Cancer	Presented Outcomes	CAD status	Cancer Status	Follow-up duration
Velders et al. [39]	2013	Netherlands	Multicenter prospective observational study	208	3,215	In-hospital all-cause mortality, in-hospital cardiovascular mortality, 1-year all-cause mortality, 1-year cardiovascular mortality, in-hospital cardiogenic shock	Only patients with ACS	Active + Prior history	1-year
Hess et al. [14]	2015	USA	Observational cohort study	496	14,512	Long-term all-cause mortality, long-term cardiovascular mortality, long-term recurrent MI	ACS + Stable CAD	Active + Prior history	14-years
Wang et al. [52]	2016	USA	Retrospective observational cohort study	261	1,313	In-hospital all-cause mortality, in-hospital cardiovascular mortality, long-term all-cause mortality, in-hospital bleeding, in-hospital cardiogenic shock, in-hospital heart failure	Only patients with ACS	Active + Prior history	median 6.2 (4.3–8.5) years
Landes et al. [18]	2017	Israel	Retrospective cohort study	969	969	1-year all-cause mortality, long-term all-cause mortality, in-hospital cardiovascular mortality, 1-year cardiovascular mortality	ACS + Stable CAD	Active + Prior history	mean 6.4 ± 5.9 years
Nakatsuma et al. [48]	2018	Japan	Multicenter observational retrospective registry study	1,109	11,071	1-year bleeding, 1-year all-cause mortality, long-term all-cause mortality, long-term cardiovascular mortality, long-term bleeding, 1-year cardiovascular mortality, long-term stroke, long-term heart failure, long-term recurrent MI	ACS + Stable CAD	Active + Prior history	median 5.3 (4.6–6.1) years
Iannaccone et al. [15]	2018	Europe	Multicenter observational retrospective study	858	13,773	1-year all-cause mortality, in-hospital bleeding, 1-year bleeding, in-hospital heart failure, in-hospital recurrent MI, 1-year recurrent MI	Only patients with ACS	Active + Prior history	1-year
Tabata et al. [19]	2018	Japan	Retrospective observational cohort study	179	824	1-year all-cause mortality, 1-year cardiovascular mortality	ACS + Stable CAD	Active + Prior history	1-year
Potts et al. [51]	2019	USA	Retrospective observational cohort study	484,694	6,086,339	In-hospital all-cause mortality, in-hospital bleeding, in-hospital stroke	ACS + Stable CAD	Active + Prior history	in-hospital
Jacobs et al. [43]	2019	USA	Retrospective single-center cohort study	58	551	In-hospital all-cause mortality, in-hospital bleeding, in-hospital stroke, in-hospital cardiogenic shock, in-hospital heart failure, in-hospital recurrent MI	Only patients with ACS	Active + Prior history	in-hospital, 30 days
Borovac et al. [40]	2019	USA	Retrospective observational cohort study	18,052	7,101,487	In-hospital all-cause mortality, in-hospital stroke, in-hospital bleeding,	ACS + Stable CAD	Active + Prior history	in-hospital
Ueki et al. [38]	2019	Switzerland	Prospective cohort study	1,368	12,279	1-year all-cause mortality, long-term all-cause mortality, long-term bleeding, 1-year cardiovascular mortality, 1-year bleeding, 1-year stroke, 1-year recurrent MI	ACS + Stable CAD	Active + Prior history	1-year
Potts et al. [50]	2020	USA	Retrospective observational cohort study	15,789	6,545,656	In-hospital all-cause mortality, in-hospital stroke, in-hospital bleeding	ACS + Stable CAD	Active + Prior history	in-hospital
Nozaka et al. [49]	2020	Japan	Retrospective observational cohort study	50	1,245	Long-term all-cause mortality, long-term cardiovascular mortality, long-term heart failure, long-term recurrent MI	Only patients with ACS	Active + Prior history	median 3.8 (1.7–6.6) years
Iglesias-Garriz et al. [36]	2020	Spain	Prospective cohort observational study	53	864	Long-term all-cause mortality	Only patients with ACS	Prior history	median 643 (258–1015) days
Kwok et al. [45]	2021	UK	Retrospecitve cohort study	51,289	1,750,056	In-hospital stroke, in-hospital cardiogenic shock	ACS + Stable CAD	Active + Prior history	90 days, periprocedural
Takeuchi et al. [37]	2021	Japan	Prospective multicenter observational study	462	3,037	Long-term all-cause mortality, long-term cardiovascular mortality, long-term stroke, long-term recurrent MI,long-term heart failure	Only patients with ACS	Active + Prior history	5 years
Guo et al. [41]	2021	USA	Observational retrospective cohort study	416	768	In-hospital all-cause mortality, long-term all-cause mortality, long-term cardiovascular mortality, long-term bleeding, in-hospital stroke, in-hospital cardiogenic shock, in-hospital heart failure, in-hospital recurrent MI, long-term recurrent MI	96.5 % ACS	Active + Prior history	median 6.2 (4.2–9.0) years Cancer Patients, median 5.1 (3.6–8.2) years non-Cancer Patients
Kanenawa et al. [44]	2021	Japan	Retrospective cohort study	185	1,118	1-year all-cause mortality, 1-year cardiovascular mortality, 1-year bleeding, 1-year stroke, 1-year recurrent MI	ACS + Stable CAD	Active + Prior history	1-year
Mohamed et al. [47]	2021	USA	Retrospective cohort study	7,516	104,222	In-hospital all-cause mortality, in-hospital bleeding, in-hospital stroke	Only patients with ACS	Only Active	in-hospital
Matsumoto et al. [46]	2021	Japan	Retrospective observational cohort study	114	789	In-hospital all-cause mortality, long-term all-cause mortality, long-term bleeding, long-term cardiovascular mortality, in-hospital bleeding, in-hospital stroke, long-term heart failure, in-hospital recurrent MI	Only patients with ACS	Active + Prior history	mean 853 days
Hayashi et al. [42]	2022	Japan	Retrospective cross-sectional study	79	633	Long-term all-cause mortality, long-term bleeding, long-term stroke, long-term recurrent MI	Only patients with ACS	Prior history	median 1088 days

ACS: acute coronary syndrome, CAD: coronary artery disease, MI: myocardial infarction

### 2.2 Eligibility criteria

Study inclusion criteria were as follows: (1) studies reporting on adults (older than 18 years) undergoing PCI; (2) comparing the outcomes of patients with cancer (prior history or active) with a control group (no cancer history); (3) reporting of all-cause mortality and/or cardiovascular mortality (primary outcomes) in different time points (in-hospital, 1-year, long-term follow-up); (4) or reporting of at least one of the secondary outcomes (stroke, bleeding, recurrent myocardial infarction (MI), cardiogenic shock, heart failure) in the different time points.

### 2.3 Selection process

Two investigators conducted the screening process independently and any discrepancy was resolved through a third author. Titles and abstracts of all retrieved articles were screened, and potentially eligible articles were subsequently collected. Subsequently, the selected articles underwent full-text evaluation to determine their inclusion in the review. All articles that did not satisfy the inclusion criteria were excluded.

### 2.4 Data extraction and risk of bias assessment

Data extraction was conducted independently by two authors, with any discrepancies resolved by a third investigator. The extracted data included baseline characteristics, total sample sizes, and the number of events for each outcome. We also extracted data on in-hospital outcomes, which included events occurring during or immediately after PCI, while events beyond the one-year mark were analyzed as long-term outcomes. In the case of both matched and unmatched analyses from one study, data from propensity-matched analyses were preferred. Additionally, when event data were not explicitly reported in the studies, we extracted data from Kaplan-Meier curves using WebPlotDigitizer program [29]. Data available in previous *meta*-analyses on the same topic were also extracted. To assess the quality and potential biases of the included studies, two separate researchers used the “Risk Of Bias In Non-randomized Studies − of Interventions” (ROBINS-I) tool, and any conflict was resolved through consensus with all authors [30].

### 2.5 Statistical analysis

Statistical analysis was performed in RStudio (“meta” and “metafor” packages) [31]. Categorical variables were expressed as counts and relative frequencies, while continuous variables were expressed as means and standard deviations. The measure of effect for binary outcomes was expressed as the risk ratio (RR) with corresponding 95 % confidence intervals (CIs). A random-effects model (Mantel–Haenzel procedure) was employed to calculate the pooled RR [32]. As a complementary analysis, absolute risk differences (ARDs) were pooled using random-effects model and are being reported per 100 patients with 95 % CIs. The degree of heterogeneity among the studies was assessed using the I2 statistic and Cochran's Q test. Heterogeneity was categorized as low (I2 ≤ 25 %), moderate (I2 ≤ 50 %), or high (I2 > 50 %) [33]. Publication bias was evaluated through funnel plot inspection, along with Egger’s and Begg’s test [34,35]. Sensitivity analyses were also conducted using the leave-one-out method, to account for influential studies. Meta-regression analysis on outcomes with at least 10 studies was also conducted to explore clinical heterogeneity by including age, and publication year as covariates. The level of statistical significance was defined by a two-sided p-value threshold of 0.05.

## 3 Results

### 3.1 Study selection

**Fig. 1:**
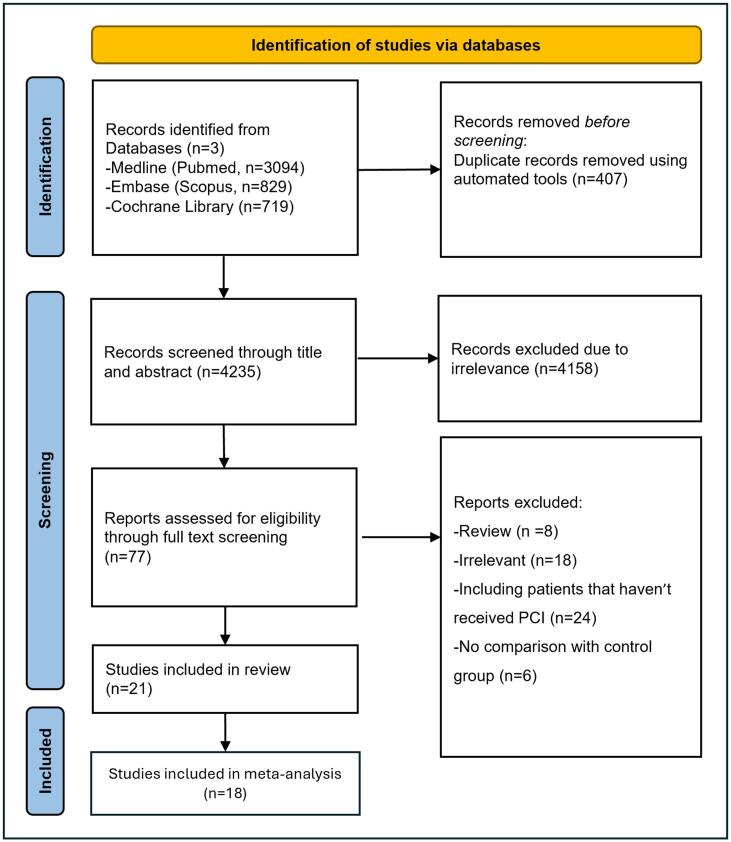
PRISMA flowchart for study selection.

The study selection process is presented in the PRISMA flowchart in Fig. 1. An initial search of the literature from all 3 databases resulted in 4,642 records. After the exclusion of duplicates, 77 articles were assessed for eligibility through full-text screening with 21 studies meeting the inclusion criteria for the systematic review. Among the included studies, 4 were prospective cohort studies [[36], [37], [38], [39]], while 17 [14,15,18,19,[40], [41], [42], [43], [44], [45], [46], [47], [48], [49], [50], [51], [52]] were retrospective studies. From the 21 studies, 7 were from Japan, 8 were from the USA, and 6 were from different European countries. A detailed description of the included studies is presented in Table 1.

### 3.2 Qualitative synthesis

In comparison with patients without cancer, patients with active or prior history of cancer were older with more comorbidities such as hypertension, diabetes mellitus, hyperlipidemia and previous myocardial infarction. A more comprehensive presentation of the baseline patient characteristics is presented in Table 2.

**Table 2:** Baseline patient characteristics of the studies included in the *meta*-analysis.

	Velders et al.[39]		Hess et al.[14]		Wang et al.[52]		Landes et al.[18]		Nakatsuma et al.[48]		Iannaccone et al.[15]		Tabata et al.[19]		Potts et al.[51]		Jacobs et al.[43]	
Group (n)	Cancer (208)	Non Cancer (3,215)	Cancer (496)	Non Cancer (14,512)	Cancer (261)	Non Cancer (1,313)	Cancer (969)	Non Cancer (969)	Cancer (1,109)	Non Cancer (11,071)	Cancer (858)	Non Cancer (13,773)	Cancer (179)	Non Cancer (824)	Cancer (484,694)	Non Cancer (6,086,339)	Cancer (58)	Non Cancer (551)
**Characteristics, n (%)**																		
Age (years) mean ± SD, median (IQR)	69.6 ± 11.0	62.8 ± 12.4	68 (61, 75)	62 (53, 71)	71.9 ± 10.7	71.7 ± 10.9	76.6 ± 10.1	76.9 ± 9.2	73.2 ± 8.5	67.8 ± 11.1	70.8 ± 10.3	62.8 ± 12.6	68.0–80.0	62.0–77.0	71 [64,78]	64 [55,73]	63.6 ± 11.7	59.4 ± 11.5
Men	141 (67.8)	2,427 (75.5)	354 (71.4)	9,586 (66.1)	177 (67.8)	890 (67.8)	700 (72.2)	700 (72.2)	825 (74)	7,976 (72)	612 (71.3)	10,632 (77.2)	86 (75.4)	410 (69.8)	316,822 (65.3)	4,041,329 (66.4)	33 (56.9)	423 (76.8)
Hypertension	88 (42.7)	1,135 (35.4)	334 (67.3)	9,478 (65.3)	197 (75.5)	986 (75.1)	843 (87)	843 (87)	904 (82)	9,100 (82)	558 (65.0)	7,961 (57.8)	84 (73.7)	460 (78.4)	351,397 (72.5)	4,230,006 (69.5)	40 (69.0)	338 (61.3)
Diabetes Mellitus	23 (11.1)	361 (11.3)	129 (26)	4,013 (27.7)	185 (70.9)	1,010 (77.0)	318 (45.7)	318 (45.7)	440 (40)	4,154 (38)	246 (28.7)	3,237 (23.5)	54 (47.4)	324 (55.2)	152,251 (31.4)	2,032,837 (33.4)	20 (34.5)	143 (26.0)
Hyperlipidemia	46 (22.4)	738 (23.1)	n/a	n/a	44 (16.9)	275 (20.9)	n/a	n/a	n/a	n/a	n/a	n/a	73 (64.0)	443 (75.5)	61,903 (12.8)	821,656 (13.5)	33 (56.9)	286 (51.9)
Chronic kidney disease	17 (8.3)	109 (3.4)	2 (2.4)	254 (1.8)	n/a	n/a	n/a	n/a	n/a	n/a	55 (6.4)	399 (2.9)	58 (50.9)	255 (43.4)	49,531 (10.2)	572,115 (9.4)	n/a	n/a
Smoking	61 (31.0)	1,487 (46.8)	238 (48)	7,712 (53.1)	168 (64.4)	819 (62.4)	230 (23.7)	204 (21)	230 (21)	3,648 (33)	n/a	n/a	9 (7.9)	109 (18.6)	169,601 (35.0)	2,160,650 (35.5)	24 (41.4)	240 (43.6)
Prior MI	35 (17.0)	335 (10.4)	246 (49.6)	7,414 (51.1)	44 (16.9)	225 (17.1)	n/a	n/a	119 (11)	1,141 (10)	132 (15.4)	1,584 (11.5)	24 (21.1)	79 (13.5)	70,609 (14.5)	797,310 (13.1)	10 (17.2)	89 (16.2)
Prior PCI	21 (10.2)	267 (8.3)	n/a	n/a	55 (21.1)	245 (18.7)	n/a	n/a	n/a	n/a	125 (14.6)	1,708 (12.4)	n/a	n/a	99,959 (20.6)	1,138,145 (18.7)	14 (24.1)	108 (19.6)
Prior CABG	5 (2.4)	79 (2.5)	n/a	n/a	32 (12.3)	134 (10.2)	185 (19.1)	196 (20.2)	n/a	n/a	40 (4.7)	427 (3.1)	n/a	n/a	38,619 (7.9)	444,303 (7.3)	7 (12.1)	20 (3.6)
Prior Stroke/TIA	24 (11.7)	191 (5.9)	66 (13.3)	1,196 (8.2)	37 (14.2)	140 (10.7)	87 (9)	63 (6.5)	142 (13)	1,149 (10)	71 (8.3)	744 (5.4)	14 (12.3)	114 (19.4)	28,305 (5.8)	225,195 (3.7)	9 (15.5)	31 (5.6)
Heart Failure	n/a	n/a	99 (20.2)	2,191 (15.4)	4 (1.5)	25 (1.9)	114 (11.8)	134 (13.8)	222 (20)	2,183 (20)	46 (5.4)	399 (2.9)	n/a	n/a	7,505 (1.5)	54,777 (0.9)	1 (1.7)	33 (6.0)
**Discharge Treatment**																		
Beta-blockers	160 (84.2)	2,812 (90.5)	447 (90.1)	13,077 (90.1)	247 (94.5)	1,224 (93.3)	n/a	n/a	294 (27)	3,410 (31)	640 (74.6)	11,197 (81.3)	84 (73.7)	425 (72.4)	n/a	n/a	54 (100)	504 (97.7)
ACE/ARBS	139 (73.2)	2,220 (71.5)	418 (84.3)	10,992 (75.8)	185 (70.8)	1,004 (76.5)	n/a	n/a	571 (51)	6,573 (59)	606 (70.6)	10,344 (75.1)	74 (64.9)	401 (68.3)	n/a	n/a	42 (77.8)	388 (75.2)
Statins	171 (90.0)	2,897 (93.2)	n/a	n/a	n/a	n/a	n/a	n/a	487 (44)	5,816 (53)	780 (90.9)	12,878 (93.5)	108 (94.7)	549 (93.5)	n/a	n/a	53 (98.2)	508 (98.5)
Aspirin	187 (98.4)	3,097 (99.3)	489 (98.6)	14,355 (98.9)	252 (96.7)	1,291 (98.3)	n/a	n/a	1,092 (98)	10,936 (99)	850 (99.1)	13,690 (99.4)	n/a	n/a	n/a	n/a	54 (100)	516 (100)
P2Y12 inhibitors	186 (97.9)	3,058 (98.0)	437 (88.1)	11,023 (76.0)	247 (94.5)	1,267 (96.5)	n/a	n/a	1,090 (98)	10,964 (99)	766 (89.3)	11,996 (87.1)	n/a	n/a	n/a	n/a	53 (98.1)	513 (99.4)
SD: standard deviation, IQR: interquartile range, MI: myocardial infarction, PCI: percutaneous coronary intervention, CABG: coronary artery bypass grafting, TIA: transient ischemic attack, ACE: angiotensin converting enzyme inhibitors, ARB: angiotensin receptor blocker. Data are presented as total numbers (n), percentage (%), mean ± SD, or median (IQR)																		

	Ueki et al.[38]		Nozaka et al.[49]		Iglesias-Garriz et al.[36]		Kwok et al.[45]		Takeuchi et al.[37]		Guo et al.[41]		Kanenawa et al.[44]		Matsumoto et al.[46]		Hayashi et al.[42]	
Group (n)	Cancer (1,368)	Non Cancer (12,279)	Cancer (50)	Non Cancer (1,245)	Cancer (53)	Non Cancer (864)	Cancer (51,289)	Non Cancer (1,750,056)	Cancer (462)	Non Cancer (3,037)	Cancer (416)	Non Cancer (768)	Cancer (185)	Non Cancer (1,118)	Cancer (114)	Non Cancer (789)	Cancer (79)	Non Cancer (633)
**Characteristics, n (%)**																		
Age (years) mean ± SD, median (IQR)	72.9±9.8	67.1±12.1	74±9	66±13	73.4±11.5	65.2±13.8	70 ± 11	64 ± 12	72.3 ± 8.5	64.3 ± 11.5	72.5±9.8	72.3±9.7	75.5±9.1	70.5±11.8	74.5 ± 8.5	66.0 ± 12.3	77.0±7.5	68.7±12.8
Men	977 (71.4)	9,121 (74.3)	38 (76)	982 (79)	39 (73.6)	635 (73.5)	36,466 (71.1)	1,183,037 (67.6)	339 (73)	2,375 (78)	279 (67.1)	519 (67.6)	128 (69.2)	752 (67.3)	48 (74)	614 (78)	51 (64.6)	494 (78.0)
Hypertension	1,057 (77.3)	8,350 (68.3)	39 (78)	906 (73)	26 (49.1)	399 (46.2)	37,953 (74.0)	1,303,791 (74.5)	283 (63)	1,692 (57)	323 (77.6)	607 (79.0)	156 (84.3)	882 (78.9)	45 (69)	527 (67)	49 (62.0)	361 (57.0)
Diabetes Mellitus	342 (25.0)	2,793 (22.8)	26 (52)	607 (49)	15 (28.3)	149 (17.2)	17,694 (34.5)	651,020 (37.2)	149 (33)	986 (33)	116 (27.9)	187 (24.3)	74 (40.0)	428 (38.3)	27 (42)	297 (38)	50 (63.3)	462 (73.0)
Hyperlipidemia	915 (66.9)	7,869 (64.5)	36 (72)	1,001 (80)	17 (32.1)	318 (36.8)	32,773 (63.9)	1,251,290 (71.5)	155 (35)	1,312 (45)	321 (77.2)	595 (77.5)	120 (64.9)	706 (63.1)	35 (53.9)	490 (62)	34 (43.0)	310 (49.0)
Chronic kidney disease	516 (37.9)	2,630 (23.8)	n/a	n/a	n/a	n/a	10,155 (19.8)	227,507 (13.0)	n/a	n/a	57 (13.7)	103 (13.4)	86 (46.5)	466 (41.7)	n/a	n/a	n/a	n/a
Smoking	251 (18.3)	3,377 (27.8)	14 (28)	519 (42)	14 (26.4)	328 (38.0)	25,696 (50.1)	721,023 (41.2)	n/a	n/a	57 (13.7)	103 (13.4)	21 (11.4)	192 (17.2)	16 (25)	285 (36)	25 (31.6)	240 (37.9)
Prior MI	268 (19.6)	2,050 (16.7)	4 (8)	122 (10)	n/a	n/a	7,693 (15.0)	248,507(14.2)	47 (10)	323 (11)	73 (17.5)	161 (21.0)	n/a	n/a	4 (6)	45 (6)	n/a	n/a
Prior PCI	347 (25.4)	2,678 (21.9)	6 (12)	105 (8)	41 (77.4)	740 (85.6)	10,821 (21.1)	365,761 (20.9)	n/a	n/a	24 (5.8)	45 (5.9)	n/a	n/a	n/a	n/a	n/a	n/a
Prior CABG	169 (12.4)	1,201 (9.8)	0 (0)	17 (1)	n/a	n/a	3,898 (7.6)	136,504 (7.8)	n/a	n/a	69 (16.6)	127 (16.5)	n/a	n/a	n/a	n/a	n/a	n/a
Prior Stroke/TIA	140 (10.2)	850 (6.9)	n/a	n/a	6 (1.1)	19 (2.2)	3,949 (7.7)	117,254 (6.7)	n/a	n/a	43 (10.3)	79 (10.3)	20 (10.8)	104 (9.3)	n/a	n/a	n/a	n/a
Heart Failure	n/a	n/a	n/a	n/a	n/a	n/a	n/a	n/a	n/a	n/a	76 (18.3)	130 (16.9)	21 (11.4)	89 (8.0)	n/a	n/a	n/a	n/a
**Discharge Treatment**																		
Beta-blockers	n/a	n/a	n/a	n/a	29 (54.7)	536 (62.0)	n/a	n/a	280 (66)	1,482 (49)	346 (86.5)	654 (85.6)	n/a	n/a	62 (66.7)	506 (78)	59 (74.7)	481 (76.0)
ACE/ARBs	n/a	n/a	n/a	n/a	n/a	n/a	n/a	n/a	n/a	n/a	249 (62.2)	465 (60.9)	n/a	n/a	72 (77.4)	572 (88)	70 (88.6)	567 (89.6)
Statins	1,170 (85.8)	11,062 (90.8)	n/a	n/a	n/a	n/a	n/a	n/a	231 (57)	1,373 (46)	224 (88.2)	485 (90.3)	86 (46.5)	525 (47.0)	82 (88.1)	601 (92)	72 (91.1)	582 (91.9)
Aspirin	1,318 (96.3)	11,901 (97.0)	n/a	n/a	42 (79.2)	699 (80.9)	n/a	n/a	n/a	n/a	385 (96.2)	742 (97.1)	180 (97.3)	1,104 (98.7)	n/a	n/a	n/a	n/a
P2Y12 inhibitors	1,327 (97)	12,041 (98.2)	n/a	n/a	37 (69.9)	574 (66.4)	n/a	n/a	n/a	n/a	393 (98.2)	742 (96.6)	182 (98.4)	1,108 (99.1)	n/a	n/a	n/a	n/a

D: standard deviation, IQR: interquartile range, MI: myocardial infarction, PCI: percutaneous coronary intervention, CABG: coronary artery bypass grafting, TIA: transient ischemic attack, ACE: angiotensin converting enzyme inhibitors, ARB: angiotensin receptor blocker. Data are presented as total numbers (n), percentage (%), mean ± SD, or median (IQR)

Out of the studies included in the systematic review, data concerning in-hospital outcomes were available in 12 studies [15,18,[39], [40], [41],^43^,[45], [46], [47],[50], [51], [52]], while data for 1-year and long-term clinical outcomes were displayed in 7 [15,18,19,38,39,44,48] and 11 [14,18,[36], [37], [38],^41^,^42^,^46^,^48^,^49^,^52^] studies respectively. Long‑term follow‑up ranged from approximately 1.7 to 14 years across included studies.

All-cause mortality rates were reported in 20 [14,15,18,19,[36], [37], [38], [39], [40], [41], [42], [43], [44],[46], [47], [48], [49], [50], [51], [52]] of the included studies, with 17 [14,15,18,[36], [37], [38], [39], [40], [41], [42],[46], [47], [48], [49], [50], [51], [52]] studies indicating a higher risk in the cancer groups in one or more of the analyzed time points. Additionally, cardiovascular mortality was present in 12 studies [14,18,19,[37], [38], [39],^41^,^44^,^46^,^48^,^49^,^52^]. However, only 5 studies demonstrated an association between fatal cardiovascular events and cancer comorbidity [18,38,39,46,48]. Cardiovascular complications were also present in most of the included studies. Bleeding events were displayed in 13 [15,38,[40], [41], [42], [43], [44],[46], [47], [48],[50], [51], [52]] studies, where patients with active or prior history of cancer were associated with higher rates in 10 [15,38,[40], [41], [42],[46], [47], [48],^50^,^51^] of them across all time points. Furthermore, among the 13 studies analyzing stroke events [37,38,[40], [41], [42], [43], [44], [45], [46], [47], [48],^50^,^51^], a significant association between active or prior history of cancer and stroke was observed in only 7 studies [37,40,42,45,47,50,51]. Cardiogenic shock was present in 5 studies [39,41,43,45,52], of which only Kwok et al. identified an association between the occurrence of new events and cancer [45]. This study included cardiogenic shock events during PCI procedure. Heart failure and recurrent MI events were present in 8 [15,37,41,43,46,48,49,52] and 11 [14,15,37,38,[41], [42], [43], [44],^46^,^48^,^49^] studies respectively.

Among the included studies, outcomes specifically related to patients with acute coronary syndrome (ACS) were reported in 11 studies [15,36,37,39,[41], [42], [43],^46^,^47^,^49^,^52^]. Lastly, in 6 out of 21 studies patients were stratified based on their cancer status (active cancer, prior history of cancer or time of cancer diagnosis) [14,39,41,45,46,51]. Additionally, two studies focused exclusively on patients with a prior history of cancer [36,42], while one study included only those with active cancer [47].

### 3.3 Risk of bias assessment

All studies were evaluated and considered as having moderate risk of bias due to confounding and missing data. The outcomes of the risk of bias assessment using the ROBINS-I evaluation system are presented in the (Supplemental Fig. 1). Publication bias was also assessed using funnel plots (Supplemental Figs. 2-4) paired with Egger’s and Begg’s tests (Supplemental Tables 2-3). There was no significant evidence of asymmetry or publication bias across the analyses, except for in-hospital cardiogenic shock, where Begg’s test indicated potential bias (p = 0.0143). However, this finding was not replicated by Egger’s test (p = 0.0881), suggesting that the pooled outcome was likely unaffected by a missing study. Notably, the small number of included studies in each outcome significantly limits the reliability of both the statistical and visual assessments of publication bias.

**Fig. 2:**
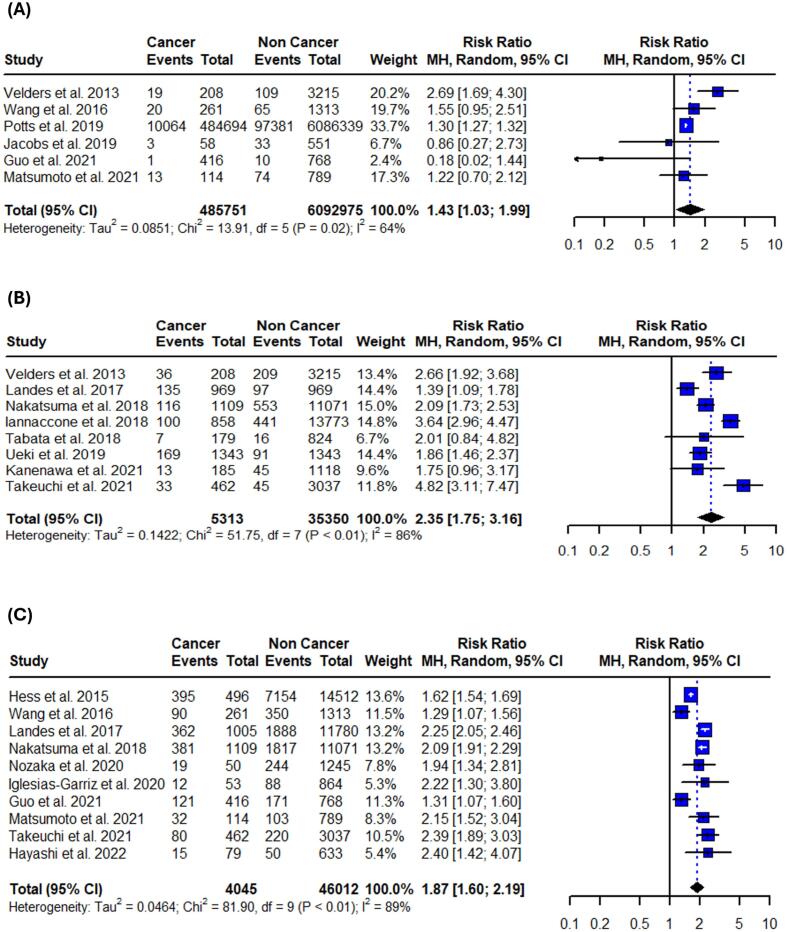
Forest plots of all-cause mortality outcomes in patients with active or prior history of cancer following PCI. (A) In-hospital all-cause mortality. (B) 1-year all-cause mortality. (C) Long-term all-cause mortality. Relative risks were calculated using random effects model, and are presented as squares, while 95% confidence intervals are being displayed as horizontal lines.

**Fig. 3:**
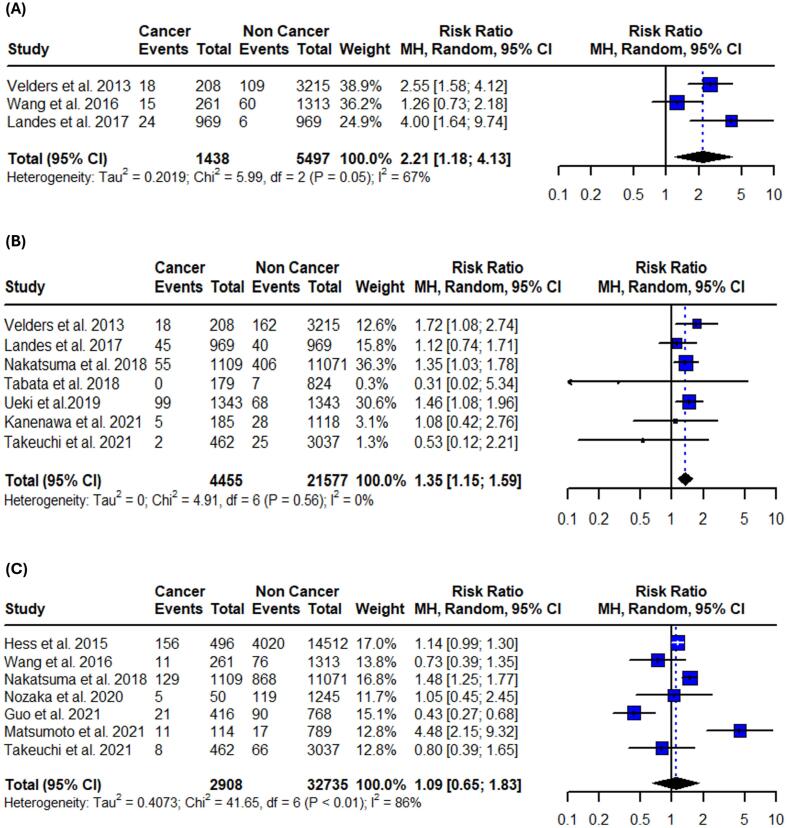
Forest plots of cardiovascular mortality outcomes in patients with active or prior history of cancer following PCI. (A) In-hospital cardiovascular mortality. (B) 1-year cardiovascular mortality. (C) Long-term cardiovascular mortality. Relative risks were calculated using random effects model, and are presented as squares, while 95% confidence intervals are being displayed as horizontal lines.

**Fig. 4:**
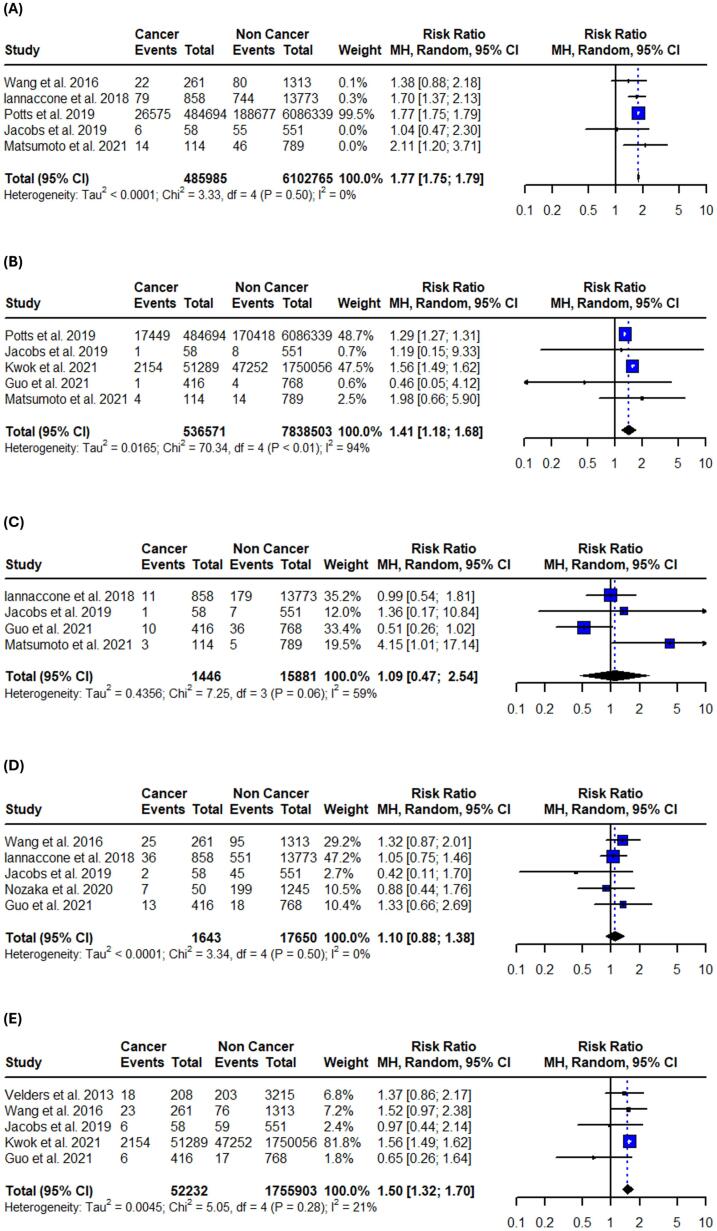
Forest plots of in-hospital adverse cardiovascular events in patients with active or prior history of cancer following PCI. (A) Bleeding. (B) Stroke. (C) Recurrent myocardial infarction. (D) Heart failure. (E) Cardiogenic shock. Relative risks were calculated using random effects model, and are presented as squares, while 95% confidence intervals are being displayed as horizontal lines.

**Table 3:** Summary of pooled risk ratios and absolute risk differences.

Outcome	RR (95 % CI)	I^2^	Control risk (95 % CI)	ARD per 100 (95 % CI)
**Primary**				
In-hospital all-cause mortality	1.43 (1.03–1.99)	64 %	3.6 % (1.8–7.1)	1.2 % (−0.9 to 3.2)
1-year all-cause mortality	2.35 (1.75–3.16)	86 %	4.2 % (3.1–5.8)	5.5 % (3.9 to 7.1)
Long-term all-cause mortality	1.87 (1.60–2.19)	89 %	16.7 % (9.7–27.3)	15.2 % (10.3 to 20.1)
In-hospital CV mortality	2.21 (1.18–4.13)	67 %	2.5 % (1.4–4.6)	2.0 % (0.9 to 3.1)
1-year CV mortality	1.35 (1.15–1.59)	0 %	2.8 % (1.9–4.0)	0.6 % (−0.4 to 1.5)
Long-term CV mortality	1.09 (0.65–1.83)	86 %	7.1 % (3.2–14.8)	0.8 % (−2.7 to 4.2)
**Secondary**				
In-hospital bleeding	1.77 (1.75–1.79)	0 %	5.6 % (3.9–8.1)	2.7 % (1.8 to 3.6)
In-hospital stroke	1.41 (1.18–1.68)	94 %	1.8 % (1.1–3.0)	0.8 % (−0.1 to 1.6)
In-hospital recurrent MI	1.09 (0.47–2.54)	59 %	1.6 % (0.7–3.6)	−0.2 % (−1.8 to 1.4)
In-hospital heart failure	1.10 (0.88–1.38)	0 %	6.3 % (3.3–11.8)	0.3 % (−0.8 to 1.4)
In-hospital cardiogenic shock	1.50 (1.32–1.70)	21 %	4.8 % (2.7–8.3)	1.0 % (−0.5 to 2.6)
1-year bleeding	1.63 (1.26–2.11)	69 %	5.8 % (3.3–9.8)	3.33 % (2.1 to 4.6)
1-year stroke	1.90 (1.21–3.00)	0 %	1.0 % (0.6–1.8)	0.9 % (0.2 to 1.6)
1-year recurrent MI	1.12 (0.55–2.25)	90 %	2.3 % (1.0–5.3)	0.6 % (−2.0 to 3.1)
Long-term bleeding	2.08 (1.30–3.35)	80 %	6.4 % (3.9–10.2)	6.3 % (1.8 to 10.7)
Long-term stroke	1.75 (0.96–3.19)	81 %	3.1 % (1.5–6.4)	1.6 % (0.5 to 3.7)
Long-term recurrent MI	1.05 (0.64–1.70)	86 %	4.8 % (2.5–8.9)	0.3 % (−3.3 to 3.8)
Long-term heart failure	1.45 (1.25–1.70)	0 %	6.3 % (5.2–7.6)	3.0 % (1.5 to 4.4)

ARD: absolute risk difference, CV: cardiovascular, MI: myocardial infarction.

### 3.4 Quantitative synthesis

After the exclusion of 3 studies due to overlapping cohort [40,47,50], 18 studies finally included in the *meta*-analysis encompassing a total of 8,446,204 patients, including 542,848 with active or prior history of cancer and 7,903,356 without a history of cancer.

#### 3.4.1 Primary outcomes

Patients with active or prior history of cancer treated with PCI faced a 43 % higher risk of in-hospital mortality (95 % CI: 1.03–––1.99; I^2^ = 64 %;), 135 % increased risk for 1-year all-cause mortality (95 % CI: 1.75 – 3.16; I^2^ = 86 %;) and 87 % higher risk for long-term all-cause mortality (95 % CI: 1.60–––2.19; I^2^ = 89 %;) when compared to patients without cancer (Fig. 2, Table 3). Sensitivity analyses using the leave-one-out method did not reveal statistically significant changes in the 1-year and long-term results. However, in the in-hospital setting, the results were not significant after omitting the studies by Wang et al., Potts et al., and Matsumoto et al. (Supplemental Fig. 5). The test for subgroup differences between studies that included only patients with ACS and those that included both ACS and stable CAD revealed significance in 1-year all-cause mortality rates (p < 0.01) (Supplemental Fig. 10).

**Fig. 5:**
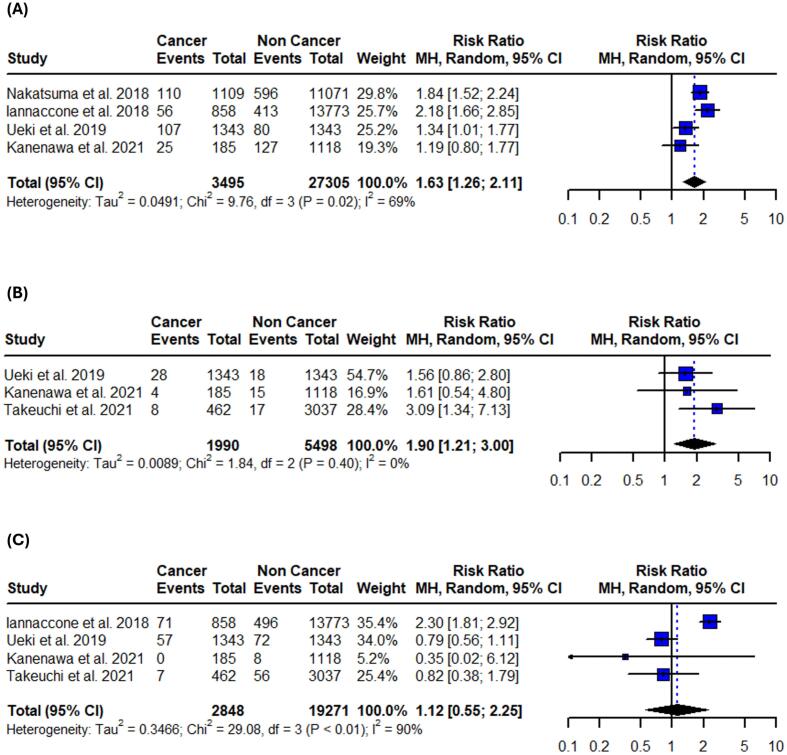
Forest plots of 1-year adverse cardiovascular outcomes in patients with active or prior history of cancer after PCI. (A) Bleeding. (B) Stroke. (C) Recurrent myocardial infarction. Relative risks were calculated using random effects model, and are presented as squares, while 95% confidence intervals are being displayed as horizontal lines.

Patients with active or prior history of cancer treated with PCI had also 121 % higher risk for in-hospital cardiovascular mortality (95 %CI: 1.18–4.13; I^2^ = 67 %;) and 35 % higher risk for 1-year cardiovascular mortality (95 % CI: 1.15–1.59; I^2^ = 0 %;) when compared to patients without cancer. No significance was observed in long-term cardiovascular mortality (RR: 1.09; 95 %CI: 0.65–1.83; I^2^ = 86 %;) (Fig. 3, Table 3). Long-term and 1-year results remained unaffected after the leave-one-out sensitivity analysis. However, in-hospital cardiovascular mortality was not significant after omitting the studies by Velders et al., and Landes et al. (Supplemental Fig. 6). Subgroup analysis revealed no differences between studies that included only patients with ACS and those that also included stable CAD (Supplemental Fig. 11).

**Fig. 6:**
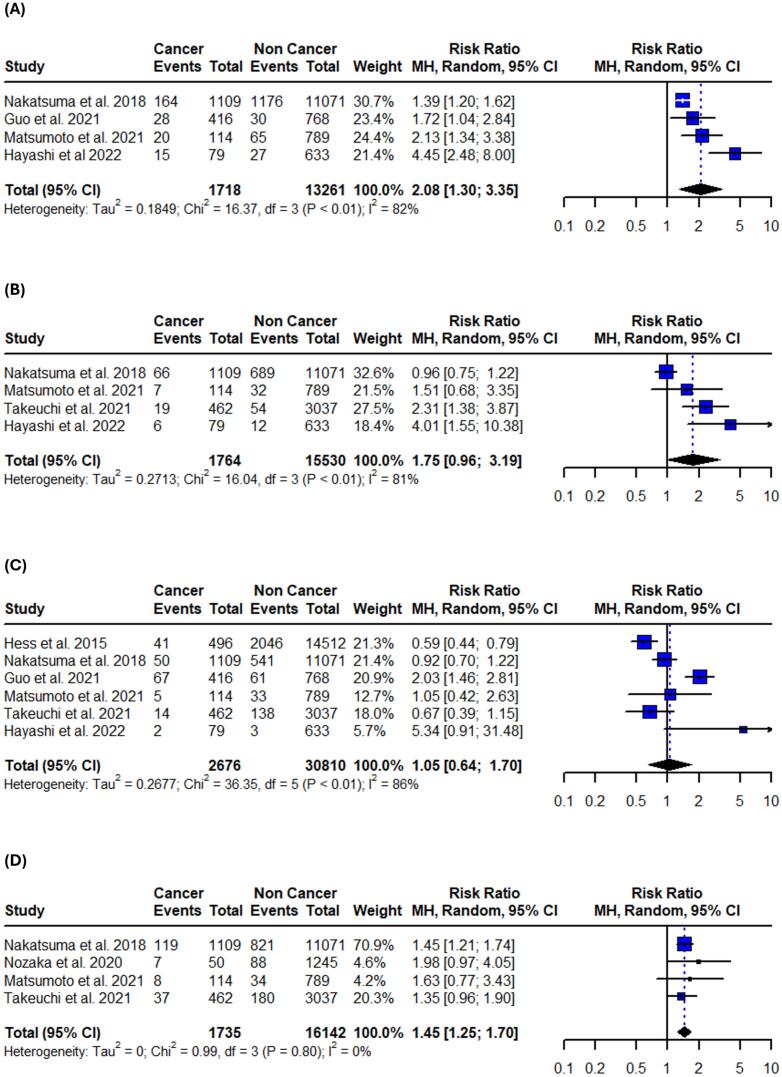
Forest plots of long-term adverse cardiovascular outcomes in patients with active or prior history of cancer after PCI. (A) Bleeding. (B) Stroke. (C) Recurrent myocardial infarction. (D) Heart failure. Relative risks were calculated using random effects model, and are presented as squares, while 95% confidence intervals are being displayed as horizontal lines.

#### 3.4.2 Secondary outcomes

***In-hospital***

Patients with active or prior history of cancer demonstrated increased in-hospital secondary clinical outcomes after PCI in all different time points. They were associated with increased in-hospital bleeding events (RR: 1.77; 95 % CI: 1.75 – 1.79; I^2^ = 0 %;), in-hospital stroke events (RR: 1.41; 95 % CI: 1.18 – 1.68; I^2^ = 94 %;), and in-hospital cardiogenic shock events (RR: 1.50; 95 % CI: 1.32 – 1.70; I^2^ = 21 %;). No statistical differences were found in in-hospital heart failure (RR: 1.10; 95 %CI: 0.88 – 1.38; I^2^ = 0 %;), and recurrent MI rates (RR: 1.09; 95 %CI: 0.47–––2.54; I^2^ = 59 %;) (Fig. 4, Table 3). Sensitivity analyses using the leave-one-out method were performed and the results remained unaffected for the majority of the in-hospital outcomes. Regarding cardiogenic shock the results were not significant after omitting the study by Kwok et al. (Supplemental Fig. 7). Subgroup analysis revealed no differences, suggesting that the impact of cancer on in-hospital cardiovascular outcomes is similar regardless of ACS status (Supplemental Fig. 12).

***1-year***.

Patients with active or prior history of cancer treated with PCI have higher risk for 1-year bleeding events (RR: 1.63; 95 %CI: 1.26 – 2.11; I^2^ = 69 %;) and 1-year stroke events (RR: 1.90; 95 %CI:1.21 – 3.00; I^2^ = 0 %;). Concurrently, recurrent MI events (RR: 1.12; 95 %CI: 0.55 – 2.25; I^2^ = 90 %;) were not significantly different between the two groups (Fig. 5, Table 3). The results remained unaffected after the leave-one-out sensitivity analysis in both bleeding and recurrent MI events. However, 1-year stroke events were not significant after omitting the study by Takeuchi et al. (Supplemental Fig. 8). The test for subgroup differences between studies that included only patients with ACS and those that included both ACS and stable CAD revealed borderline significance in 1-year bleeding events (p = 0.05) (Supplemental Fig. 13).

***Long-term:***

Patients with active or prior history of cancer treated with PCI were significantly associated with long-term bleeding events (RR: 2.08; 95 % CI: 1.30 – 3.35; I^2^ = 82 %;), in addition to heart failure events (RR: 1.45; 95 % CI: 1.25 – 1.70; I^2^ = 0 %;). No significant difference in long-term stroke events (RR: 1.75; 95 %CI: 0.96 – 3.19; I^2^ = 81 %;), and recurrent MI events (RR: 1.05; 95 %CI: 0.64 – 1.70; I^2^ = 86 %;) was observed (Fig. 6, Table 3). Sensitivity analyses using the leave-one-out method didn’t affect the significance of the results in most of the long-term outcomes. Stroke events were significantly higher in the active or prior history of cancer group after omitting the study Nakatsuma et al. (Supplemental Fig. 9). Subgroup analysis revealed that studies that included only patients with ACS demonstrate higher rates of both bleeding (p = 0.04) and stroke (p < 0.01) events (Supplemental Fig. 14).

### 3.5 Meta-regression analysis

Meta-regression for mean age of the study population and the year of publication were reported in more than 10 studies and could serve as covariates. The subsequent *meta*-regression analyses showed that neither mean age nor publication year significantly influenced the effect estimates (p = 0.62 and p = 0.19, respectively). Adjusting for these covariates did not meaningfully alter the effect sizes or reduce the observed heterogeneity. Visual inspection of the *meta*-regression plot revealed a positive trend, indicating that RR tends to increase with more recent publication years, but this was not statistically significant (Supplemental Figs. 15,16).

## 4 Discussion

The present systematic review and *meta*-analysis highlights the impact of cancer on adverse event outcomes in patients undergoing PCI. In the present analysis of 18 studiesactive or prior history of cancer was associated with an increased mortality risk, as well as increased risk for bleeding, stroke, cardiogenic shock, and heart failure events.

Prior systematic reviews and *meta*-analyses have demonstrated that compared to individuals without a history or evidence of cancer, patients with active or prior history of cancer have a significant increase in mortality rates [25,26]. The present study supports these results as active or prior history of cancer was associated with increased in-hospital and 1-year all-cause mortality, while also adding information for the long-term negative impact of cancer on mortality rates in this population. Higher mortality rates may be attributed to a more complicated clinical profile among cancer patients, including a higher prevalence of comorbidities like hypertension, diabetes, and hyperlipidemia, which may complicate their recovery following PCI [53]. Moreover, it has been established that cancer pathophysiology and chemotherapeutic agents can exacerbate cardiovascular disease through mechanisms of accelerated atherosclerosis and direct myocardial damage [9,10].

Furthermore, in the present study in-hospital and 1-year cardiovascular mortality were higher in patients with active or prior history of cancer, a result not replicated for long-term cardiovascular mortality. This may be attributed to the cumulative effect of cancer and its treatment over time. Cancer progression may have a more prominent role in the late mortality of these patients. However, large epidemiological data evaluating emergency department cardiovascular disease (CVD) presentations demonstrated that cancer status was associated with higher risk of mortality in all CVD categories [54].

In a recent analysis of multicenter controlled high-bleeding risk trials Campos et al. documented that cancer patients treated with DAPT are at increased risk of developing the 1-year net adverse clinical event (HR; 1.25; p = 0.01), mostly driven by all-cause mortality and major bleeding events [55]. In our analysis, patients with active or prior history of cancer were associated withan increased rate of in-hospital, 1-year and long-term bleeding events. Bleeding complications may derive from the altered coagulation status that is associated with malignancy itself, which can increase both thrombotic and bleeding events [56]. Moreover, chemotherapy-induced thrombocytopenia may further increase the risk of bleeding [57]. Anticoagulation and antiplatelet therapy have additional challenges in this high-risk patient population. Optimal agent selection and DAPT duration requires further evaluation to minimize both thrombotic and hemorrhagic risk [58].

In a large population-based cohort study, Mulder et al. demonstrated that cancer patients are at increased risk of developing arterial thromboembolism, including stroke [59]. Moreover, insights from the OACIS registry displayed that patients with active or prior history of cancer have increased risk of long-term stroke events after PCI (HR: 2.40; 95 %CI: 1.41–4.08). However, this result that was not replicated after adjusting for confounding factors (HR: 1.41; 95 %CI: 0.67–2.97) [37]. The results of the present analysis showed a higher rate of in-hospital and 1-year stroke events in the cancer population after PCI. This increased risk of stroke events could be related to the prothrombotic state associated with malignancy and possible effects of cancer treatments such as small vessel disease and intravascular coagulopathy [60]. Furthermore, in the present study a higher rate of in-hospital cardiogenic shock was reported among patients with active or prior history of cancer. This may be a result of their compromised cardiac function due to cancer therapies and a higher burden of comorbidities [61]. Moreover, the *meta*-analysis results demonstrated that long-term heart failure events were more frequent in patients with active or prior history of cancer, when compared to in-hospital results. It emphasizes the chronic nature of cardiac dysfunction in this population, usually a result of the cardiotoxic effects of many chemotherapeutic agents and radiation therapy over time [58].

In the studies by Iannaccone et al. and Guo et al., patients with active or prior history of cancer were associated with higher risk of 1-year and long-term recurrent MI events after PCI respectively [15,41]. In contrast, the present analysis demonstrated no significant differences in recurrent MI rates between the two groups at any time point. DAPT duration differences between the studies may be an explanation for these findings. Therefore, additional studies are needed to explore the possible risk factors for recurrent MI in this population.

Following the 2022 ESC guidelines on cardio-oncology long-term follow-up on cancer survivors focuses more on risk-factor reduction and lifestyle modification [62]. In the present analysis patients with active or prior history of cancer demonstrated higher rates of long-term mortality, in addition to cardiovascular complications. Consequently, long-term monitoring due to the increased rate of adverse cardiovascular outcomes derived from the cumulative effect of cancer and its treatment over time becomes evident. Cardiovascular trends from the CDC WONDER dataset demonstrated a 52 % age-adjusted mortality rate reduction. These findings highlight the impact of multidisciplinary management and cardio oncology services have made in patients with active or prior history of cancer [63].

Although all studies were deemed as having a moderate risk of bias according to the ROBINS-I evaluation system, several domains could have influenced the effect size of certain outcomes. Specifically, residual confounding such as cancer stage, ACS severity, anemia/thrombocytopenia, and antithrombotic treatment selection/duration may have influenced early adverse events (e.g. in-hospital mortality, bleeding, cardiogenic shock). Additionally, higher early mortality rates as a competing risk in patients with active or prior history of cancer could have reduced the number of long-term non-fatal events (MI, stroke).

In the subgroup analysis, tests revealed significant results in 1-year all-cause mortality and bleeding events, in addition to long term stroke events. These findings suggest that the characteristics of patients with ACS, such as high thrombotic risk, use of more portent P2Y12 inhibitors, and longer DAPT duration, may amplify mortality rates and cardiovascular events in patients with active or prior history of cancer after PCI.

Lastly, our findings suggest that the differences in outcomes between patients with and without cancer undergoing PCI cannot be attributed solely to the average age of the studied populations or the era in which the research was conducted. Instead, other factors, whether clinical, methodological, or related to treatment approaches, may better explain the observed heterogeneity, underscoring the need for further investigation.

## 5 Study limitations

There are several limitations that must be addressed before interpreting the results of this systematic review and *meta*-analysis. Firstly, the analysis was conducted using study-level data and no adjustment for baseline patient characteristics was made, therefore introducing the potential for confounding bias. Secondly, the observational nature of the studies could further compromise the accuracy of the findings due to inherent allocation bias between the baseline characteristics that an otherwise randomized control study would have accounted for. Furthermore, studies included in long-term pooled results varied in follow-up durations. Even though we used random-effects inverse-variance models, studies were weighted by statistical power, not by duration of follow up, thus contributing to the between-study heterogeneity. Moreover, stratification based on the type of cancer was not feasible due to lack of adequate data from the included studies, further increasing the between-study heterogeneity. Additionally, despite the large overall sample size of over 8 million patients, most of the patients included came from 2 large-scale retrospective studies. Notably, not all *meta*-analyses incorporated all of the studies included, since vast majority involved merely thousands of patients per analysis.

## 6 Conclusion

This *meta*-analysis, including over 8.4 million patients, highlights that cancer comorbidity significantly worsens outcomes following PCI. Cancer was associated with increased risks of in-hospital, 1-year, and long-term all-cause mortality, as well as elevated risks of cardiovascular mortality at earlier time points. Secondary outcomes showed higher rates of bleeding, stroke, and cardiogenic shock in cancer patients during hospitalization, with persistent bleeding and heart failure risks in the long-term time point. These findings emphasize the need for tailored peri- and post-PCI management strategies to address the unique risks faced by patients with active or prior history of cancer. Further studies are warranted to evaluate the impact of specific cancer types on clinical outcomes after PCI.

## CRediT authorship contribution statement

**Nikolaos Vythoulkas-Biotis:** Writing – original draft, Methodology, Investigation, Formal analysis, Data curation, Conceptualization. **David-Dimitris Chlorogiannis:** Writing – original draft, Visualization, Methodology, Formal analysis. **Theoni Theodoropoulou:** Writing – original draft, Investigation, Data curation, Conceptualization. **Ioannis Gialamas:** Writing – review & editing, Visualization. **Evangelos Oikonomou:** Writing – review & editing. **Konstantinos Kalogeras:** Writing – review & editing. **Helena Michalopoulou:** Methodology, Conceptualization. **Gerasimos Siasos:** Supervision. **Emanuel Vavuranakis:** Supervision.

## Declaration of competing interest

The authors declare that they have no known competing financial interests or personal relationships that could have appeared to influence the work reported in this paper.

## References

[1] Dai H., Much A.A., Maor E., Asher E., Younis A., Xu Y., Lu Y., Liu X., Shu J., Bragazzi N.L. (2022). Global, regional, and national burden of ischaemic heart disease and its attributable risk factors, 1990-2017: results from the Global Burden of Disease Study 2017. Eur. Heart J. Qual. Care Clin. Outcomes.

[2] Sung H., Ferlay J., Siegel R.L., Laversanne M., Soerjomataram I., Jemal A., Bray F. (2021). Global Cancer Statistics 2020: GLOBOCAN estimates of incidence and mortality worldwide for 36 cancers in 185 countries. CA Cancer J. Clin..

[3] Siegel R.L., Giaquinto A.N., Jemal A. (2024). Cancer statistics, 2024. CA Cancer J. Clin..

[4] Zoller B., Ji J., Sundquist J., Sundquist K. (2012). Risk of coronary heart disease in patients with cancer: a nationwide follow-up study from Sweden. Eur. J. Cancer.

[5] Aunan J.R., Cho W.C., Soreide K. (2017). The biology of aging and cancer: a brief overview of shared and divergent molecular hallmarks. Aging Dis..

[6] Calle E.E., Kaaks R. (2004). Overweight, obesity and cancer: epidemiological evidence and proposed mechanisms. Nat. Rev. Cancer.

[7] Kuper H., Boffetta P., Adami H.O. (2002). Tobacco use and cancer causation: association by tumour type. J. Intern. Med..

[8] Ball S., Ghosh R.K., Wongsaengsak S., Bandyopadhyay D., Ghosh G.C., Aronow W.S., Fonarow G.C., Lenihan D.J., Bhatt D.L. (2019). Cardiovascular toxicities of immune checkpoint inhibitors: JACC review topic of the week. J. Am. Coll. Cardiol..

[9] Cuomo J.R., Javaheri S.P., Sharma G.K., Kapoor D., Berman A.E., Weintraub N.L. (2018). How to prevent and manage radiation-induced coronary artery disease. Heart.

[10] Monsuez J.J., Charniot J.C., Vignat N., Artigou J.Y. (2010). Cardiac side-effects of cancer chemotherapy. Int. J. Cardiol..

[11] Zamorano J.L., Lancellotti P., Rodriguez Munoz D., Aboyans V., Asteggiano R., Galderisi M., Habib G., Lenihan D.J., Lip G.Y.H., Lyon A.R., Lopez Fernandez T., Mohty D., Piepoli M.F., Tamargo J., Torbicki A., Suter T.M., E.S.C.S.D. Group (2016). 2016 ESC Position Paper on cancer treatments and cardiovascular toxicity developed under the auspices of the ESC Committee for Practice Guidelines: The Task Force for cancer treatments and cardiovascular toxicity of the European Society of Cardiology (ESC). Eur. Heart J..

[12] Anderson S.G., Ratib K., Myint P.K., Keavney B., Kwok C.S., Zaman A., Ludman P.F., de Belder M.A., Nolan J., Mamas M.A., S. (2015). British Cardiovascular intervention, R. National Institute for Cardiovascular Outcomes, Impact of age on access site-related outcomes in 469,983 percutaneous coronary intervention procedures: Insights from the British Cardiovascular intervention Society. Catheter. Cardiovasc. Interv..

[13] Mamas M.A., Fath-Ordoubadi F., Danzi G.B., Spaepen E., Kwok C.S., Buchan I., Peek N., de Belder M.A., Ludman P.F., Paunovic D., Urban P. (2015). Prevalence and Impact of co-morbidity burden as defined by the charlson co-morbidity index on 30-day and 1- and 5-year outcomes after coronary stent implantation (from the Nobori-2 Study). Am. J. Cardiol..

[14] Hess C.N., Roe M.T., Clare R.M., Chiswell K., Kelly J., Tcheng J.E., Hagstrom E., James S.K., Khouri M.G., Hirsch B.R., Kong D.F., Abernethy A.P., Krucoff M.W. (2015). Relationship between cancer and cardiovascular outcomes following percutaneous coronary intervention. J. Am. Heart Assoc..

[15] Iannaccone M., D'Ascenzo F., Vadala P., Wilton S.B., Noussan P., Colombo F., Raposeiras Roubin S., Abu Assi E., Gonzalez-Juanatey J.R., Simao Henriques J.P., Saucedo J., Kikkert W.J., Nunez-Gil I., Ariza-Sole A., Song X.T., Alexopoulos D., Liebetrau C., Kawaji T., Moretti C., Garbo R., Huczek Z., Nie S.P., Fujii T., Correia L.C., Kawashiri M.A., Garcia Acuna J.M., Southern D., Alfonso E., Terol B., Garay A., Zhang D., Chen Y., Xanthopoulou I., Osman N., Mollmann H., Shiomi H., Giordana F., Kowara M., Filipiak K., Wang X., Yan Y., Fan J.Y., Ikari Y., Nakahashi T., Sakata K., Gaita F., Yamagishi M., Kalpak O., Kedev S. (2018). Prevalence and outcome of patients with cancer and acute coronary syndrome undergoing percutaneous coronary intervention: a BleeMACS substudy. Eur. Heart J. Acute Cardiovasc. Care.

[16] Baber U., Mehran R., Giustino G., Cohen D.J., Henry T.D., Sartori S., Ariti C., Litherland C., Dangas G., Gibson C.M., Krucoff M.W., Moliterno D.J., Kirtane A.J., Stone G.W., Colombo A., Chieffo A., Kini A.S., Witzenbichler B., Weisz G., Steg P.G., Pocock S. (2016). Coronary thrombosis and major bleeding after PCI with drug-eluting stents: risk scores from PARIS. J. Am. Coll. Cardiol..

[17] Yeh R.W., Secemsky E.A., Kereiakes D.J., Normand S.L., Gershlick A.H., Cohen D.J., Spertus J.A., Steg P.G., Cutlip D.E., Rinaldi M.J., Camenzind E., Wijns W., Apruzzese P.K., Song Y., Massaro J.M., Mauri L., Investigators D.S. (2016). Development and validation of a prediction rule for benefit and harm of dual antiplatelet therapy beyond 1 year after percutaneous coronary intervention. J. Am. Med. Assoc..

[18] Landes U., Kornowski R., Bental T., Assali A., Vaknin-Assa H., Lev E., Iakobishvili Z. (2017). Long-term outcomes after percutaneous coronary interventions in cancer survivors. Coron. Artery Dis..

[19] Tabata N., Sueta D., Yamamoto E., Takashio S., Arima Y., Araki S., Yamanaga K., Ishii M., Sakamoto K., Kanazawa H., Fujisue K., Hanatani S., Soejima H., Hokimoto S., Izumiya Y., Kojima S., Yamabe H., Kaikita K., Tsujita K., K.S. Investigators (2018). Outcome of current and history of cancer on the risk of cardiovascular events following percutaneous coronary intervention: a Kumamoto University Malignancy and Atherosclerosis (KUMA) study. Eur. Heart J. Qual. Care Clin. Outcomes.

[20] Blann A.D., Dunmore S. (2011). Arterial and venous thrombosis in cancer patients. Cardiol. Res. Pract..

[21] Goldberg G.L., Gibbon D.G., Smith H.O., DeVictoria C., Runowicz C.D., Burns E.R. (1994). Clinical impact of chemotherapy-induced thrombocytopenia in patients with gynecologic cancer. J. Clin. Oncol..

[22] McCarthy C.P., Steg G., Bhatt D.L. (2017). The management of antiplatelet therapy in acute coronary syndrome patients with thrombocytopenia: a clinical conundrum. Eur. Heart J..

[23] Wallentin L., Becker R.C., Budaj A., Cannon C.P., Emanuelsson H., Held C., Horrow J., Husted S., James S., Katus H., Mahaffey K.W., Scirica B.M., Skene A., Steg P.G., Storey R.F., Harrington R.A., Investigators P., Freij A., Thorsen M. (2009). Ticagrelor versus clopidogrel in patients with acute coronary syndromes. N. Engl. J. Med..

[24] Wiviott S.D., Braunwald E., McCabe C.H., Montalescot G., Ruzyllo W., Gottlieb S., Neumann F.J., Ardissino D., De Servi S., Murphy S.A., Riesmeyer J., Weerakkody G., Gibson C.M., Antman E.M., Investigators T.-T. (2007). Prasugrel versus clopidogrel in patients with acute coronary syndromes. N. Engl. J. Med..

[25] Machanahalli Balakrishna A., Ismayl M., Srinivasamurthy R., Gowda R.M., Aboeata A. (2022). Early outcomes of percutaneous coronary intervention in patients with cancer: a systematic review and meta-analysis. Curr. Probl. Cardiol..

[26] Quintana R.A., Monlezun D.J., Davogustto G., Saenz H.R., Lozano-Ruiz F., Sueta D., Tsujita K., Landes U., Denktas A.E., Alam M., Paniagua D., Addison D., Jneid H. (2020). Outcomes following percutaneous coronary intervention in patients with cancer. Int. J. Cardiol..

[27] Roule V., Verdier L., Blanchart K., Ardouin P., Lemaitre A., Bignon M., Sabatier R., Alexandre J., Beygui F. (2020). Systematic review and meta-analysis of the prognostic impact of cancer among patients with acute coronary syndrome and/or percutaneous coronary intervention. BMC Cardiovasc. Disord..

[28] Page M.J., McKenzie J.E., Bossuyt P.M., Boutron I., Hoffmann T.C., Mulrow C.D., Shamseer L., Tetzlaff J.M., Akl E.A., Brennan S.E., Chou R., Glanville J., Grimshaw J.M., Hrobjartsson A., Lalu M.M., Li T., Loder E.W., Mayo-Wilson E., McDonald S., McGuinness L.A., Stewart L.A., Thomas J., Tricco A.C., Welch V.A., Whiting P., Moher D. (2021). The PRISMA 2020 statement: an updated guideline for reporting systematic reviews. BMJ.

[29] Rohatgi A. (2017). Web Plot Digitizer.

[30] Sterne J.A., Hernan M.A., Reeves B.C., Savovic J., Berkman N.D., Viswanathan M., Henry D., Altman D.G., Ansari M.T., Boutron I., Carpenter J.R., Chan A.W., Churchill R., Deeks J.J., Hrobjartsson A., Kirkham J., Juni P., Loke Y.K., Pigott T.D., Ramsay C.R., Regidor D., Rothstein H.R., Sandhu L., Santaguida P.L., Schunemann H.J., Shea B., Shrier I., Tugwell P., Turner L., Valentine J.C., Waddington H., Waters E., Wells G.A., Whiting P.F., Higgins J.P. (2016). ROBINS-I: a tool for assessing risk of bias in non-randomised studies of interventions. BMJ.

[31] Lortie C.J., Filazzola A. (2020). A contrast of meta and metafor packages for meta-analyses in R. Ecol. Evol..

[32] Fidler V., Nagelkerke N. (2013). The Mantel-Haenszel procedure revisited: models and generalizations. PLoS One.

[33] Higgins J.P., Thompson S.G., Deeks J.J., Altman D.G. (2003). Measuring inconsistency in meta-analyses. BMJ.

[34] Begg C.B., Mazumdar M. (1994). Operating characteristics of a rank correlation test for publication bias. Biometrics.

[35] Egger M., Davey Smith G., Schneider M., Minder C. (1997). Bias in meta-analysis detected by a simple, graphical test. BMJ.

[36] Iglesias-Garriz I., Delgado I., Prieto-Salvador I., Garrote C., Garcia-Palomo A., Fernandez-Vazquez F. (2020). Previously diagnosed cancer and mortality after ST-segment elevation acute myocardial infarction treated with primary angioplasty. Catheter. Cardiovasc. Interv..

[37] Takeuchi T., Hikoso S., Hattori S., Kitamura T., Nakatani D., Mizuno H., Okada K., Dohi T., Kojima T., Kida H., Sunaga A., Oeun B., Sato T., Sakata Y., Sato H., Hori M., Komuro I., Sobue T., Sakata Y., G. (2021). Osaka acute coronary insufficiency study, the effect of a cancer history on patients with acute myocardial infarction after percutaneous coronary intervention. Int. Heart J..

[38] Ueki Y., Vogeli B., Karagiannis A., Zanchin T., Zanchin C., Rhyner D., Otsuka T., Praz F., Siontis G.C.M., Moro C., Stortecky S., Billinger M., Valgimigli M., Pilgrim T., Windecker S., Suter T., Raber L. (2019). Ischemia and bleeding in cancer patients undergoing percutaneous coronary intervention. JACC CardioOncol..

[39] Velders M.A., Boden H., Hofma S.H., Osanto S., van der Hoeven B.L., Heestermans A.A., Cannegieter S.C., Jukema J.W., Umans V.A., Schalij M.J., van Boven A.J. (2013). Outcome after ST elevation myocardial infarction in patients with cancer treated with primary percutaneous coronary intervention. Am. J. Cardiol..

[40] Borovac J.A., Kwok C.S., Iliescu C., Lee H.J., Kim P.Y., Palaskas N.L., Zaman A., Butler R., Lopez-Mattei J.C., Mamas M.A. (2019). Percutaneous coronary intervention and outcomes in patients with lymphoma in the United States (Nationwide Inpatient Sample [NIS] Analysis). Am. J. Cardiol..

[41] Guo W., Fan X., Lewis B.R., Johnson M.P., Rihal C.S., Lerman A., Herrmann J. (2021). Cancer patients have a higher risk of thrombotic and ischemic events after percutaneous coronary intervention. J. Am. Coll. Cardiol. Intv..

[42] Hayashi H., Kataoka Y., Murai K., Sawada K., Iwai T., Matama H., Honda S., Fujino M., Yoneda S., Takagi K., Otsuka F., Asaumi Y., Izumiya Y., Fukuda D., Noguchi T. (2022). Cardiovascular and bleeding risks of inactive cancer in patients with acute myocardial infarction who received primary percutaneous coronary intervention using drug-eluting stent and dual/triple antithrombotic therapy. Cardiovasc. Diagn. Ther..

[43] Jacobs J.A., Pickworth K., Boudoulas K.D., Hinkley M., McLaughlin E., Blais D. (2019). Outcomes for cancer patients with acute ST-segment elevation myocardial infarction undergoing primary percutaneous coronary intervention. Cardiovasc. Revasc. Med..

[44] Kanenawa K., Yamaji K., Morinaga T., Hiromasa T., Hayashi M., Hiramori S., Tomoi Y., Kuramitsu S., Domei T., Hyodo M., Soga Y., Shirai S., Ando K. (2021). Clinical outcomes after percutaneous coronary intervention in patients with cancer. Circ J.

[45] Kwok C.S., Wong C.W., Kontopantelis E., Barac A., Brown S.A., Velagapudi P., Hilliard A.A., Bharadwaj A.S., Chadi Alraies M., Mohamed M., Bhatt D.L., Mamas M.A. (2021). Percutaneous coronary intervention in patients with cancer and readmissions within 90 days for acute myocardial infarction and bleeding in the USA. Eur. Heart J..

[46] Matsumoto T., Saito Y., Yamashita D., Sato T., Wakabayashi S., Kitahara H., Sano K., Kobayashi Y. (2021). Impact of active and historical cancer on short- and long-term outcomes in patients with acute myocardial infarction. Am. J. Cardiol..

[47] Mohamed M.O., Van Spall H.G.C., Kontopantelis E., Alkhouli M., Barac A., Elgendy I.Y., Khan S.U., Shing Kwok C., Shoaib A., Bhatt D.L., Mamas M.A. (2021). Corrigendum to: effect of primary percutaneous coronary intervention on in-hospital outcomes among active cancer patients presenting with ST-elevation myocardial infarction: a propensity score matching analysis. Eur. Heart J. Acute Cardiovasc. Care.

[48] Nakatsuma K., Shiomi H., Morimoto T., Watanabe H., Nakagawa Y., Furukawa Y., Kadota K., Ando K., Ono K., Shizuta S., Kimura T., C.-R.-K.-P.-C.-R.-C. Investigators (2018). Influence of a history of cancer on long-term cardiovascular outcomes after coronary stent implantation (an Observation from Coronary Revascularization Demonstrating Outcome Study-Kyoto Registry Cohort-2). Eur. Heart J. Qual. Care Clin. Outcomes.

[49] Nozaka M., Yokoyama H., Kitayama K., Nagawa D., Hamadate M., Miura N., Kawamura Y., Nakata M., Nishizaki F., Hanada K., Yokota T., Yamada M., Tomita H. (2020). Clinical outcomes of acute myocardial infarction patients with a history of malignant tumor. In Vivo.

[50] Potts J., Mohamed M.O., Lopez Mattei J.C., Iliescu C.A., Konopleva M., Rashid M., Bagur R., Mamas M.A. (2020). Percutaneous coronary intervention and in-hospital outcomes in patients with leukemia: a nationwide analysis. Catheter. Cardiovasc. Interv..

[51] Potts J.E., Iliescu C.A., Lopez Mattei J.C., Martinez S.C., Holmvang L., Ludman P., De Belder M.A., Kwok C.S., Rashid M., Fischman D.L., Mamas M.A. (2019). Percutaneous coronary intervention in cancer patients: a report of the prevalence and outcomes in the United States. Eur. Heart J..

[52] Wang F., Gulati R., Lennon R.J., Lewis B.R., Park J., Sandhu G.S., Wright R.S., Lerman A., Herrmann J. (2016). Cancer history portends worse acute and long-term noncardiac (but not Cardiac) mortality after primary percutaneous coronary intervention for acute ST-segment elevation myocardial infarction. Mayo Clin. Proc..

[53] Fowler H., Belot A., Ellis L., Maringe C., Luque-Fernandez M.A., Njagi E.N., Navani N., Sarfati D., Rachet B. (2020). Comorbidity prevalence among cancer patients: a population-based cohort study of four cancers. BMC Cancer.

[54] Kobo O., Moledina S.M., Raisi-Estabragh Z., Shanmuganathan J.W.D., Chieffo A., Al Ayoubi F., Alraies M.C., Biondi-Zoccai G., Elgendy I.Y., Mohamed M.O., Roguin A., Freeman P., Mamas M.A. (2022). Emergency department cardiovascular disease encounters and associated mortality in patients with cancer: a study of 20.6 million records from the USA. Int. J. Cardiol..

[55] Campos C.M., Mehran R., Capodanno D., Owen R., Windecker S., Varenne O., Stone G.W., Valgimigli M., Hajjar L.A., Kalil Filho R., Oldroyd K., Morice M.C., Urban P., Abizaid A. (2024). Risk burden of cancer in patients treated with abbreviated dual antiplatelet therapy after PCI: analysis of multicenter controlled high-bleeding risk trials. Circ. Cardiovasc. Interv..

[56] Kamphuisen P.W., Beyer-Westendorf J. (2014). Bleeding complications during anticoagulant treatment in patients with cancer. Thromb. Res..

[57] Al-Samkari H., Soff G.A. (2021). Clinical challenges and promising therapies for chemotherapy-induced thrombocytopenia. Expert Rev. Hematol..

[58] Tsigkas G., Vakka A., Apostolos A., Bousoula E., Vythoulkas-Biotis N., Koufou E.E., Vasilagkos G., Tsiafoutis I., Hamilos M., Aminian A., Davlouros P. (2023). Dual antiplatelet therapy and cancer; balancing between ischemic and bleeding risk: a narrative review. J. Cardiovasc. Dev. Dis..

[59] Mulder F.I., Horvath-Puho E., van Es N., Pedersen L., Buller H.R., Botker H.E., Sorensen H.T. (2021). Arterial thromboembolism in cancer patients: a Danish population-based cohort study. JACC CardioOncol.

[60] Bang O.Y., Chung J.W., Lee M.J., Seo W.K., Kim G.M., Ahn M.J., Investigators O.-A.-C.-S. (2020). Cancer-related stroke: an emerging subtype of ischemic stroke with unique pathomechanisms. J. Stroke.

[61] Keramida K., Parissis J.T., Chioncel O., Farmakis D. (2019). Cardiogenic shock in cancer. Heart Fail. Rev..

[62] Lyon A.R., Lopez-Fernandez T., Couch L.S., Asteggiano R., Aznar M.C., Bergler-Klein J., Boriani G., Cardinale D., Cordoba R., Cosyns B., Cutter D.J., de Azambuja E., de Boer R.A., Dent S.F., Farmakis D., Gevaert S.A., Gorog D.A., Herrmann J., Lenihan D., Moslehi J., Moura B., Salinger S.S., Stephens R., Suter T.M., Szmit S., Tamargo J., Thavendiranathan P., Tocchetti C.G., van der Meer P., van der Pal H.J.H., E.S.C.S.D. Group (2022). 2022 ESC Guidelines on cardio-oncology developed in collaboration with the European Hematology Association (EHA), the European Society for Therapeutic Radiology and Oncology (ESTRO) and the International Cardio-Oncology Society (IC-OS). Eur. Heart J..

[63] Kobo O., Khattak S., Lopez-Mattei J., Van Spall H.G.C., Graham M., Cheng R.K., Osman M., Sun L., Ullah W., Fischman D.L., Roguin A., Mohamed M.O., Mamas M.A. (2021). Trends in cardiovascular mortality of cancer patients in the US over two decades 1999-2019. Int. J. Clin. Pract..

