# Supplementary material for: Short and long-term outcomes of percutaneous coronary intervention in patients with active or prior history of cancer: a systematic review and meta-analysis
Source: Int J Cardiol Heart Vasc. 2025 Sep 22;61:101806. doi: 10.1016/j.ijcha.2025.101806 (PMC12494567; doi:10.1016/j.ijcha.2025.101806)
Supplement: Supplementary Data 1 [file mmc1.docx]

**Supplementary Material for “Short and Long-term Outcomes of Percutaneous Coronary Intervention in Patients with Active or Historical Cancer: A Systematic Review and Meta-analysis”.**

**Contents**

Search Strategy using three different databases……. ………………………………………... 2

Risk of Bias Assessment using the ROBINS-I evaluation system…………………………… 3

Mortality funnel plots………………………………………………………………………… 4

Secondary clinical outcome funnel plots……………………………………………………... 5

Secondary clinical outcome funnel plots……………………………………………………... 6

Egger’s Test for publication bias……………………………………………………………... 7

Begg’s test for publication bias………………………………………………………………. 8

Leave-one-out sensitivity analysis of all-cause mortality……………………………………. 9

Leave-one-out sensitivity analysis of cardiovascular mortality…………………………….. 10

Leave-one-out sensitivity analysis of in-hospital cardiovascular outcomes………………... 11

Leave-one-out sensitivity analysis of 1-year cardiovascular outcomes…………………….. 12

Leave-one-out sensitivity analysis of long-term cardiovascular outcomes…………………. 13

Subgroup analysis of all-cause mortality based on ACS status…………………………….. 14

Subgroup analysis of cardiovascular mortality based on ACS status ………………………. 15

Subgroup analysis of in-hospital cardiovascular outcomes based on ACS status………….. 16

Subgroup analysis of 1-year cardiovascular outcomes based on ACS status ………………. 17

Subgroup analysis of long-term cardiovascular outcomes based on ACS status…………… 18

Meta-regression analysis for long-term all-cause mortality with age………………………. 19

Meta-regression analysis for long-term all-cause mortality with publication year…………. 20

# Supplemental Table 1: Search Strategy using three different databases.

| Database Screened and Results | Search Terms | Limits Used |
| --- | --- | --- |
| Pubmed  (3,094 Results) | *((("percutaneous coronary intervention"[MeSH Terms] OR ("percutaneous"[All Fields] AND "coronary"[All Fields] AND "intervention"[All Fields]) OR "percutaneous coronary intervention"[All Fields]) AND ("coronary artery disease"[MeSH Terms] OR ("coronary"[All Fields] AND "artery"[All Fields] AND "disease"[All Fields]) OR "coronary artery disease"[All Fields])) OR ("myocardial infarction"[MeSH Terms] OR ("myocardial"[All Fields] AND "infarction"[All Fields]) OR "myocardial infarction"[All Fields])) AND ("cancer s"[All Fields] OR "cancerated"[All Fields] OR "canceration"[All Fields] OR "cancerization"[All Fields] OR "cancerized"[All Fields] OR "cancerous"[All Fields] OR "neoplasms"[MeSH Terms] OR "neoplasms"[All Fields] OR "cancer"[All Fields] OR "cancers"[All Fields]) AND ("outcome"[All Fields] OR "outcomes"[All Fields])* | English Language |
| Scopus  (829 Results) | *( TITLE-ABS-KEY ( percutaneous AND coronary AND intervention ) AND TITLE-ABS-KEY ( coronary AND artery AND disease ) OR TITLE-ABS-KEY ( myocardial AND infarction ) AND TITLE-ABS-KEY ( cancer ) AND TITLE-ABS-KEY ( outcomes ) )* | English Language |
| Cochrane Library  (719 Results) | *((((percutaneous coronary intervention) AND (coronary artery disease)) OR (myocardial infarction)) AND (cancer)) AND (outcomes)* | English Language |

*
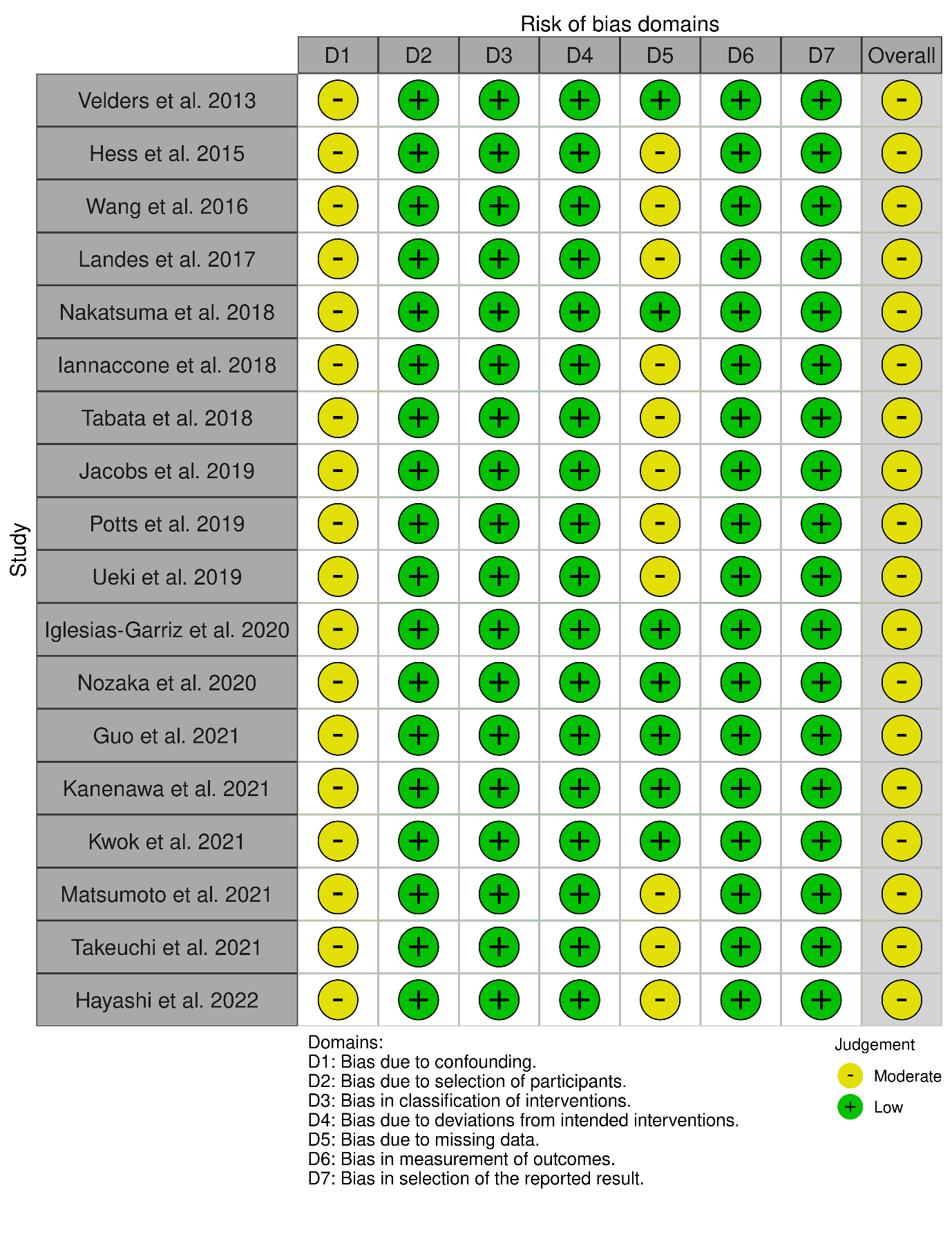
*

# Supplemental Figure 1: Risk of Bias Assessment using the ROBINS-I evaluation system.


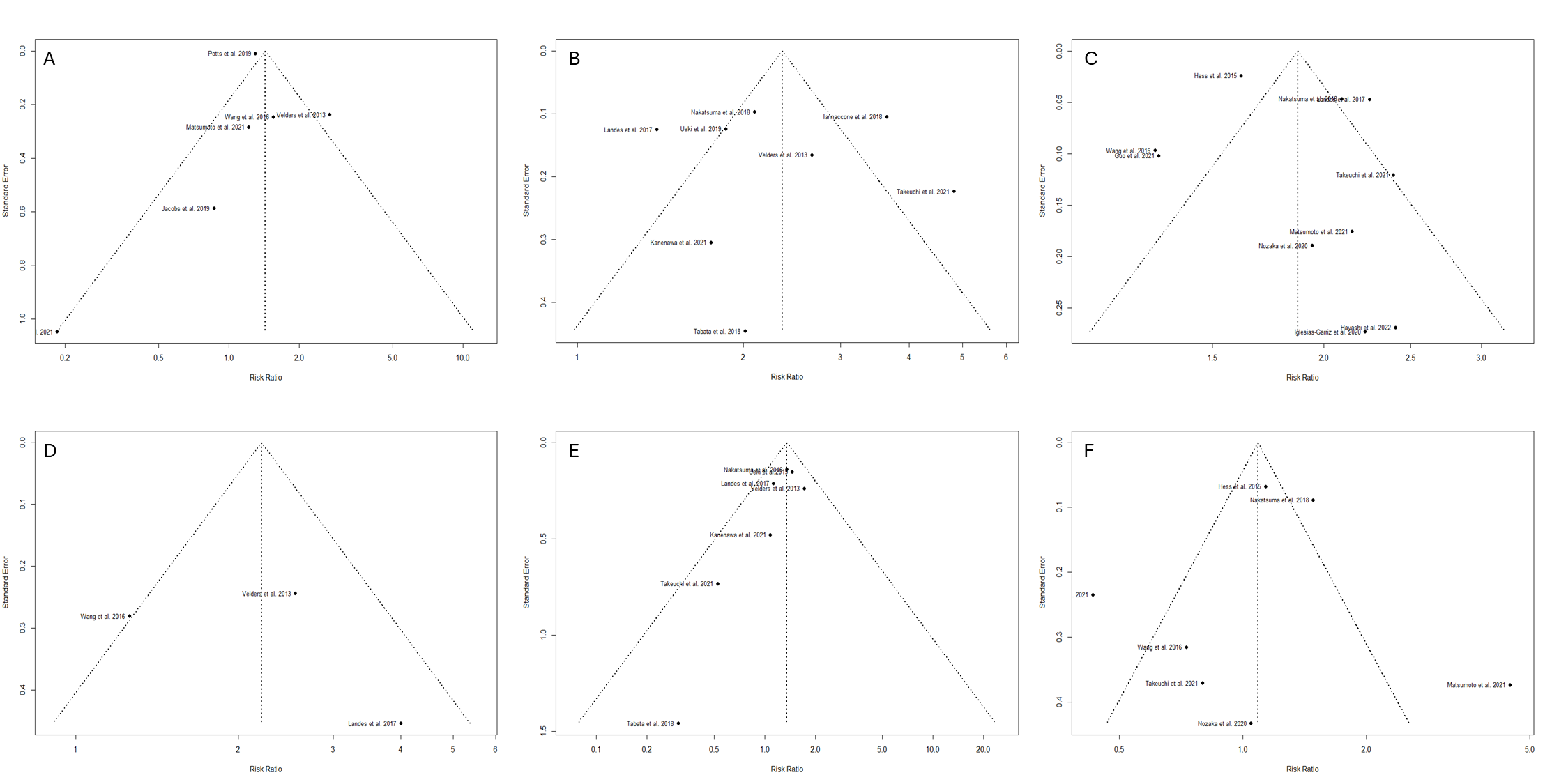


# Supplemental Figure 2: Mortality funnel plots. (A) In-hospital all-cause mortality. (B) 1-year all-cause mortality. (C) Long-term all-cause mortality. (D) In-hospital cardiovascular mortality. (E) 1-year cardiovascular mortality. (F) Long-term cardiovascular mortality


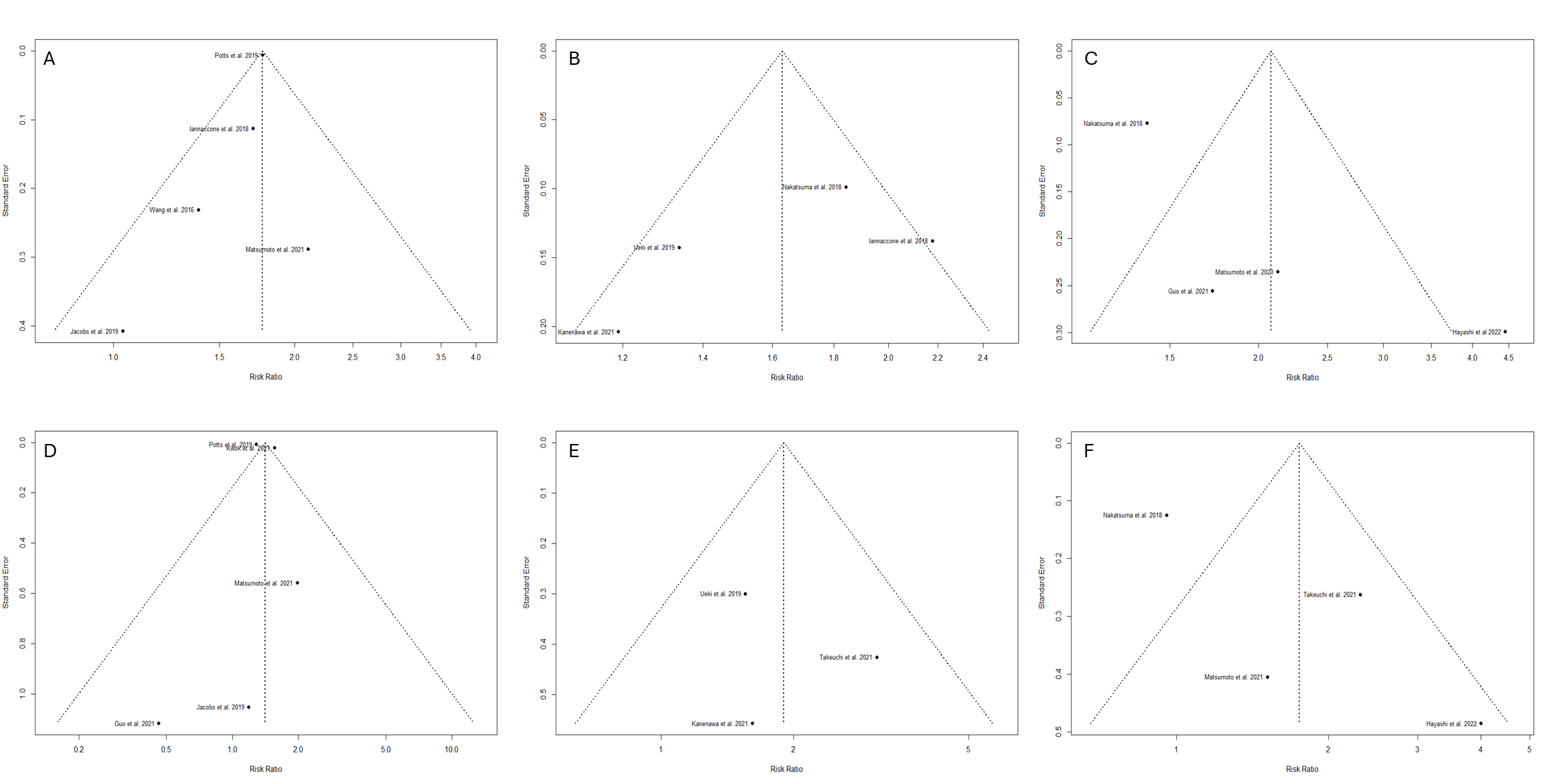


# Supplemental Figure 3: Secondary clinical outcome funnel plots. (A) In-hospital bleeding events. (B) 1-year bleeding events. (C) Long-term bleeding events. (D) In-hospital stroke events. (E) 1-year stroke events. (F) Long-term stroke events.


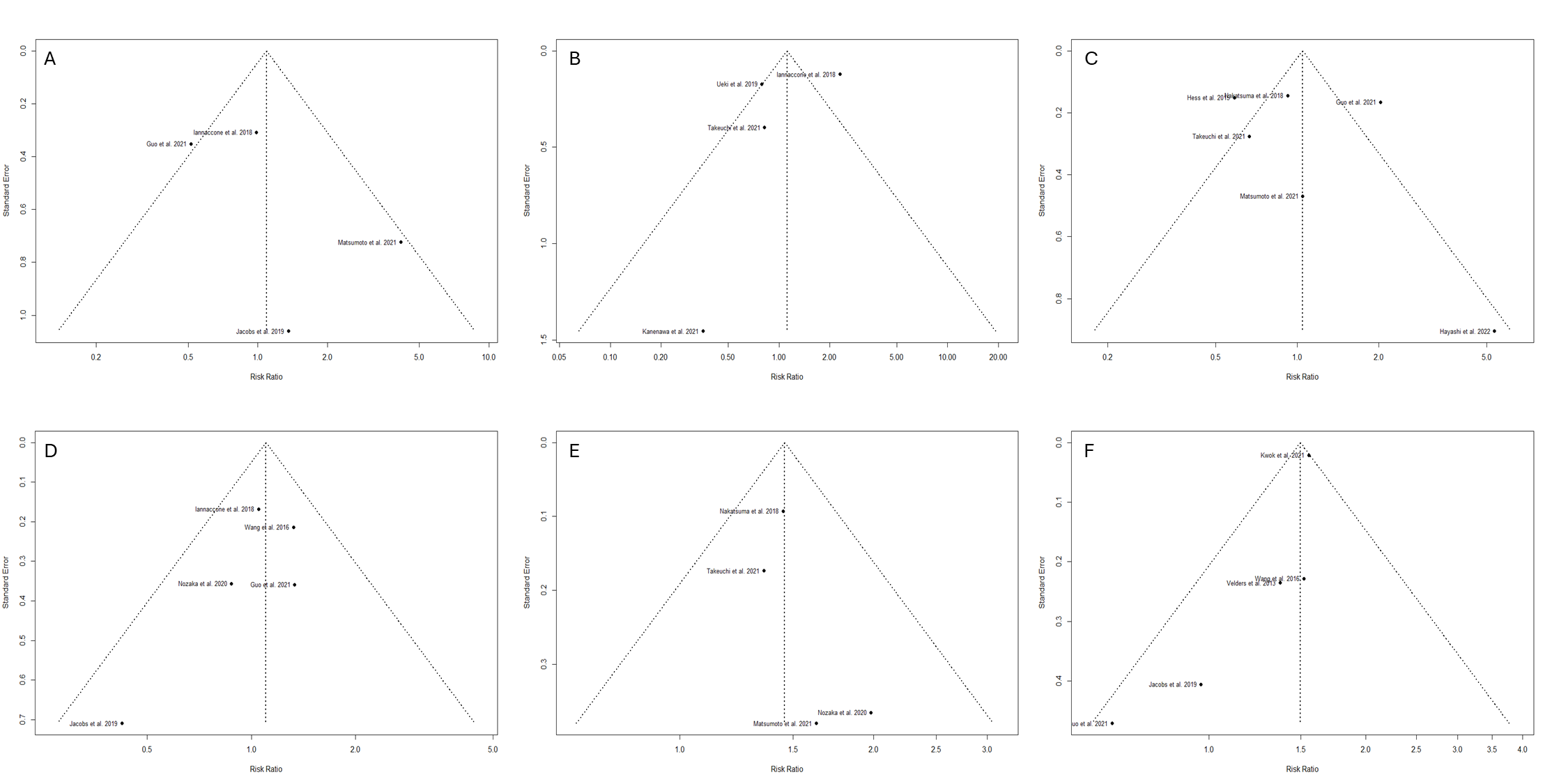


# Supplemental Figure 4: Secondary clinical outcome funnel plots. (A) In-hospital recurrent MI events. (B) 1-year recurrent MI events. (C) Long-term recurrent MI events. (D) In-hospital heart failure events. (E) Long-term heart failure events. (F) In-hospital cardiogenic shock events.

| Supplemental Table 2: Egger’s Test for publication bias | | | | | | |
| --- | --- | --- | --- | --- | --- | --- |
| **Outcome** | **t** | **df** | **p-value** | **BE** | **SE** | **Tau^2** |
| In-hospital all-cause mortality | 0.21 | 4 | 0.8418 | 0.1824 | 0.8568 | 3.4392 |
| 1-year all-cause mortality | 0.11 | 6 | 0.9132 | 0.3012 | 2.6488 | 8.6064 |
| Long-term all-cause mortality | 0.72 | 8 | 0.4933 | 1.0791 | 1.5035 | 9.6186 |
| In-hospital cardiovascular mortality | 0.47 | 1 | 0.7214 | 2.5398 | 5.4276 | 4.9134 |
| 1-year cardiovascular mortality | -2.17 | 5 | 0.0820 | -1.1180 | 0.5149 | 0.5056 |
| Long-term cardiovascular mortality | -0.42 | 5 | 0.6933 | -0.7226 | 1.7288 | 8.0490 |
| In-hospital bleeding | -1.22 | 3 | 0.3088 | -0.5432 | 0.4443 | 0.7399 |
| 1-year bleeding | -1.09 | 2 | 0.3903 | -3.8786 | 3.5659 | 3.0656 |
| Long-term bleeding | 2.35 | 2 | 0.1430 | 3.1875 | 1.3552 | 2.1728 |
| In-hospital stroke | 0.56 | 3 | 0.6150 | 1.4205 | 2.5399 | 21.2325 |
| 1-year stroke | 0.38 | 1 | 0.7677 | 1.1285 | 2.9538 | 1.6083 |
| Long-term stroke | 2.45 | 2 | 0.1342 | 3.4753 | 1.4201 | 2.0078 |
| In-hospital recurrent MI | 1.02 | 2 | 0.4142 | 1.8954 | 1.8544 | 2.3821 |
| 1-year recurrent MI | -0.87 | 2 | 0.4766 | -2,5218 | 2.9030 | 10.5556 |
| Long-term recurrent MI | 0.46 | 4 | 0.6719 | 1.2005 | 2.6317 | 8.6392 |
| In-hospital heart failure | -1.11 | 3 | 0.3491 | -1.0694 | 0.9661 | 0.7917 |
| Long-term heart failure | 1.18 | 2 | 0.3608 | 0.6144 | 0.5227 | 0.2917 |
| In-hospital cardiogenic shock | -2.49 | 3 | 0.0881 | -1.0033 | 0.4022 | 0.5474 |
| *df: degrees of freedom, BE: bias estimate, SE: standard error, MI: myocardial infarction* | | | | | | |

| Supplemental Table 3: Begg’s test for publication bias | | | | | |
| --- | --- | --- | --- | --- | --- |
| **Outcome** | **z** | **p-value** | **BE** | **SE** |  |
| In-hospital all-cause mortality | -1.32 | 0.1885 | -7.0000 | 5.3229 | |
| 1-year all-cause mortality | 0.25 | 0.8046 | 2.0000 | 8.0829 | |
| Long-term all-cause mortality | 0.27 | 0.7884 | 3.0000 | 11.1803 | |
| In-hospital cardiovascular mortality | 0.52 | 0.6015 | 1.0000 | 1.9149 | |
| 1-year cardiovascular mortality | -1.35 | 0.1765 | -9.0000 | 6.6583 | |
| Long-term cardiovascular mortality | 0.75 | 0.4527 | 5.0000 | 6.6583 | |
| In-hospital bleeding | -1.47 | 0.1416 | -6.0000 | 4.0825 | |
| 1-year bleeding | -0.68 | 0.4969 | -2.0000 | 2.9439 | |
| Long-term bleeding | 1.36 | 0.1742 | 4.0000 | 2.9439 | |
| In-hospital stroke | -0.49 | 0.6242 | -2.0000 | 4.0825 | |
| 1-year stroke | 0.52 | 0.6015 | 1.0000 | 1.9149 | |
| Long-term stroke | 0.68 | 0.4969 | 2.0000 | 2.9439 | |
| In-hospital recurrent MI | 0.00 | 1.0000 | 0.0000 | 2.9439 | |
| 1-year recurrent MI | 0.00 | 1.0000 | 0.0000 | 2.9439 | |
| Long-term recurrent MI | 0.94 | 0.3476 | 5.0000 | 5.3229 | |
| In-hospital heart failure | -0.98 | 0.3272 | -4.0000 | 4.0825 | |
| Long-term heart failure | 0.68 | 0.4969 | 2.0000 | 2.9439 | |
| In-hospital cardiogenic shock | -2.45 | 0.0143 | -10.0000 | 4.0825 | |
| *BE: bias estimate, SE: standard error, MI: myocardial infarction* | | | | | |


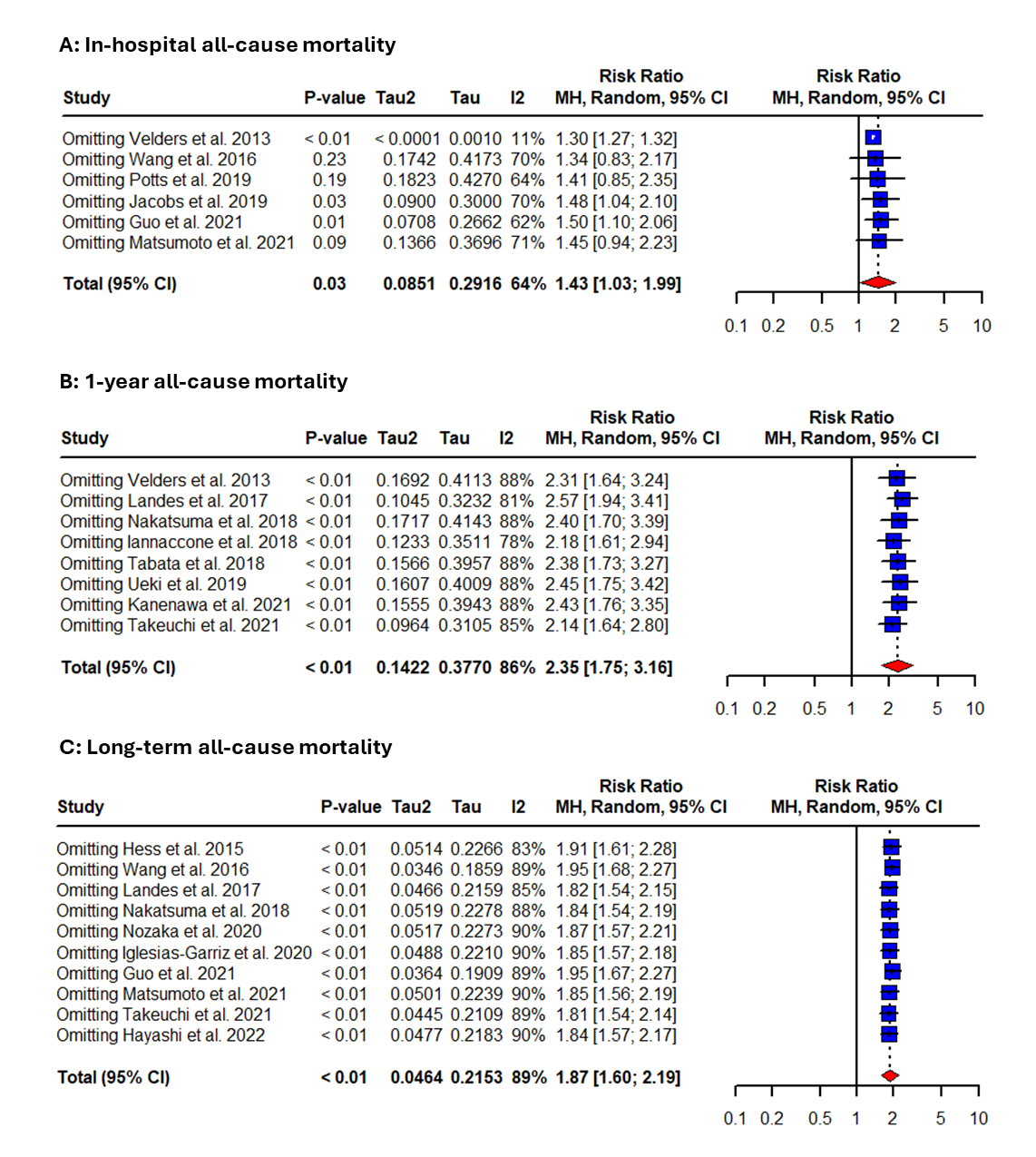


# Supplemental Figure 5: Leave-one-out sensitivity analysis of all-cause mortality by omitting each individual study. (A) In-hospital all-cause mortality. (B) 1-year all-cause mortality. (C) long-term all-cause mortality.


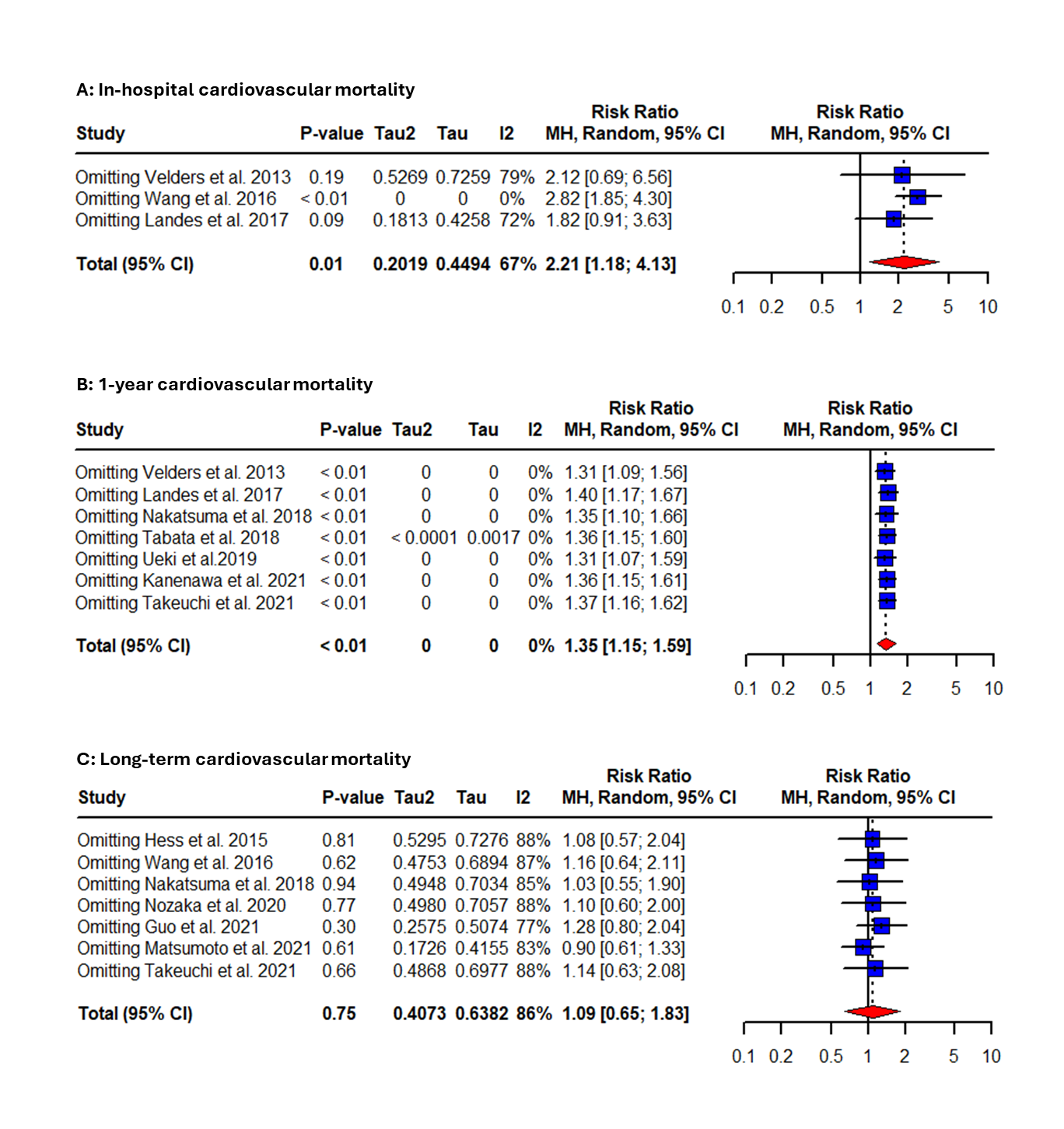


# Supplemental Figure 6: Leave-one-out sensitivity analysis of cardiovascular mortality by omitting each individual study. (A) In-hospital cardiovascular mortality. (B) 1-year cardiovascular mortality. (C) long-term cardiovascular mortality.


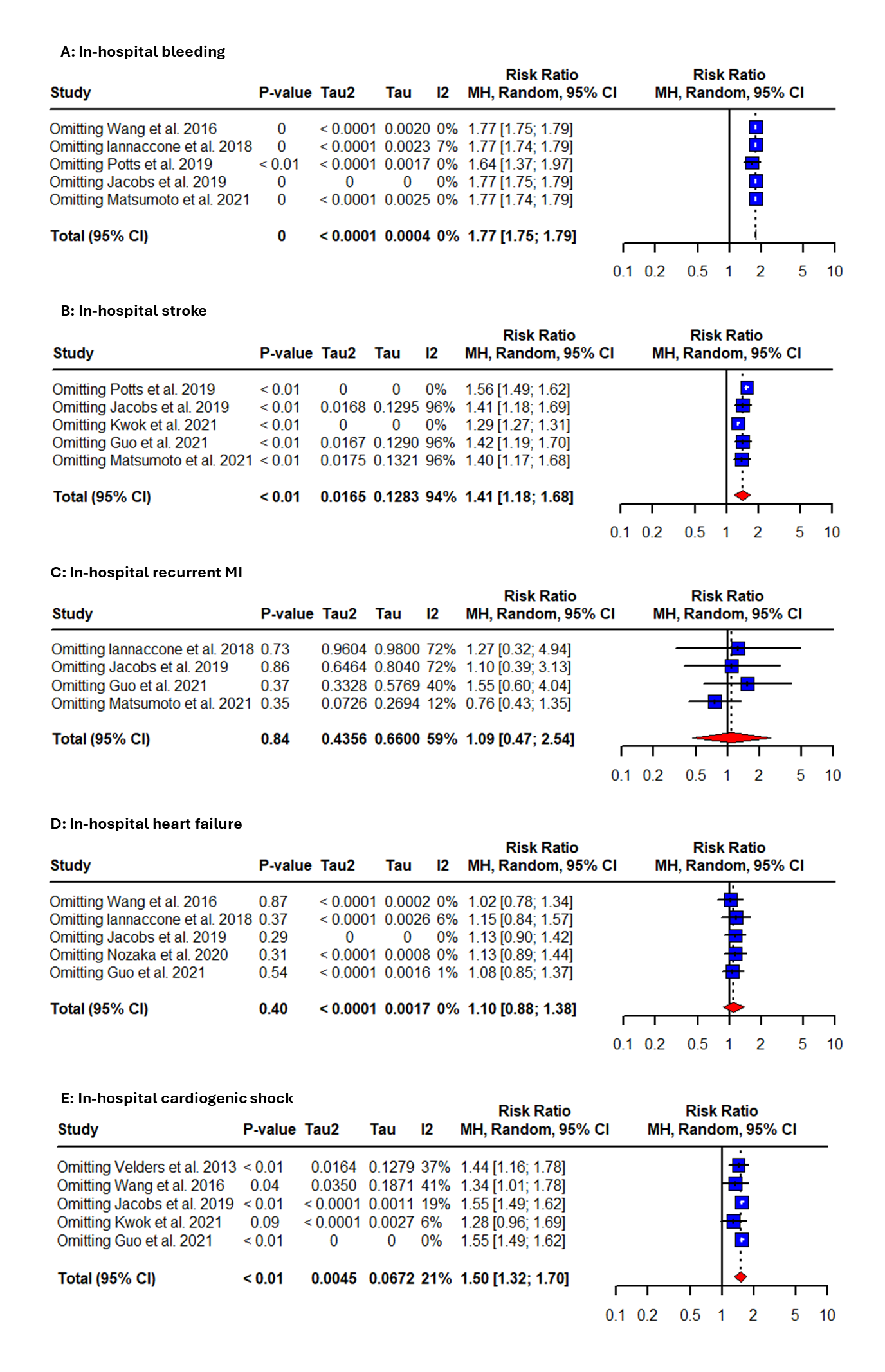


# Supplemental Figure 7: Leave-one-out sensitivity analysis of in-hospital cardiovascular outcomes by omitting each individual study. (A) in-hospital bleeding. (B) in-hospital stroke. (C) in-hospital recurrent MI. (D) in-hospital heart failure. (E) in-hospital cardiogenic shock.


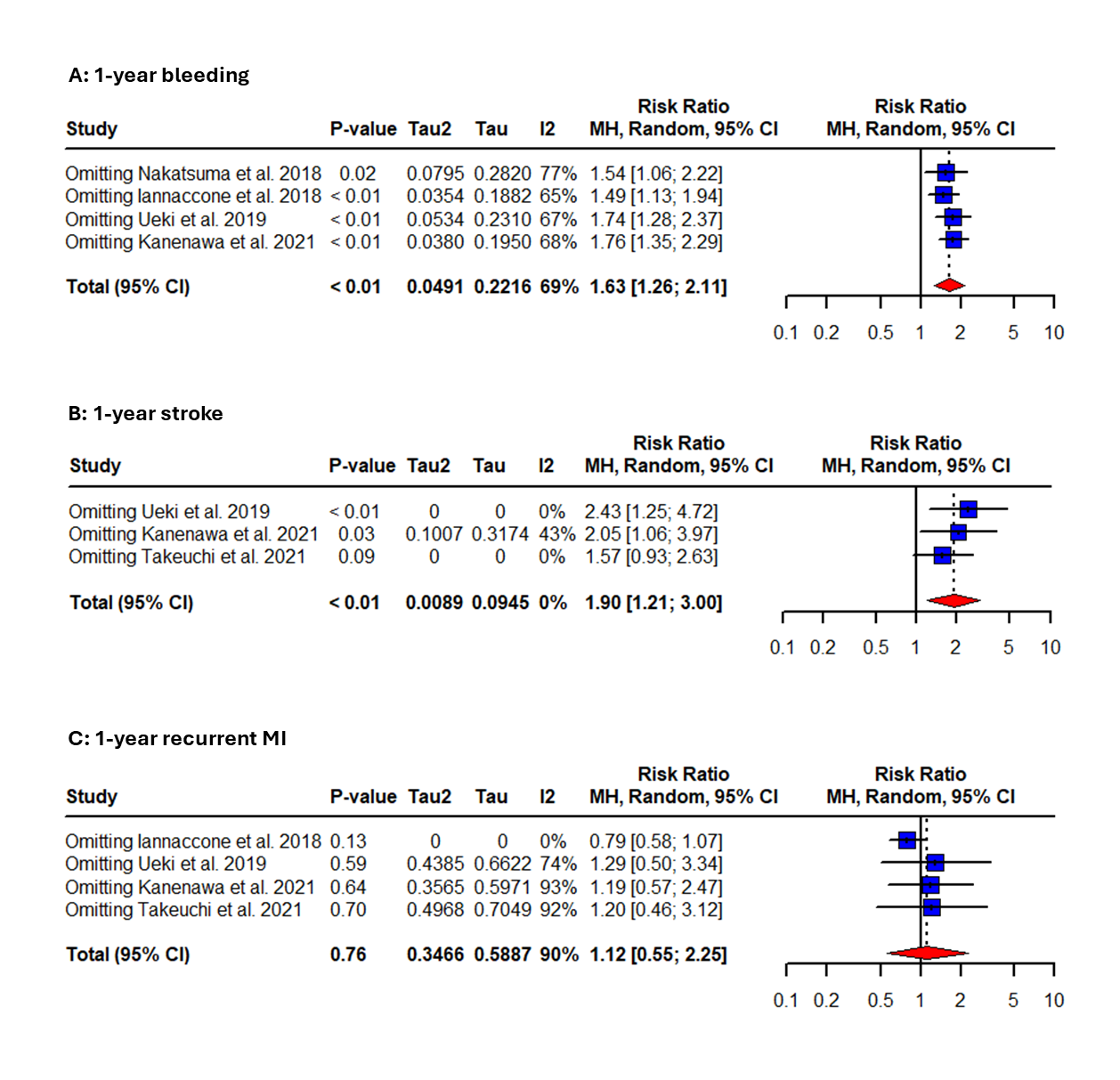


# Supplemental Figure 8: Leave-one-out sensitivity analysis of 1-year cardiovascular outcomes by omitting each individual study. (A) 1-year bleeding. (B) 1-year stroke. (C) 1-year recurrent MI.


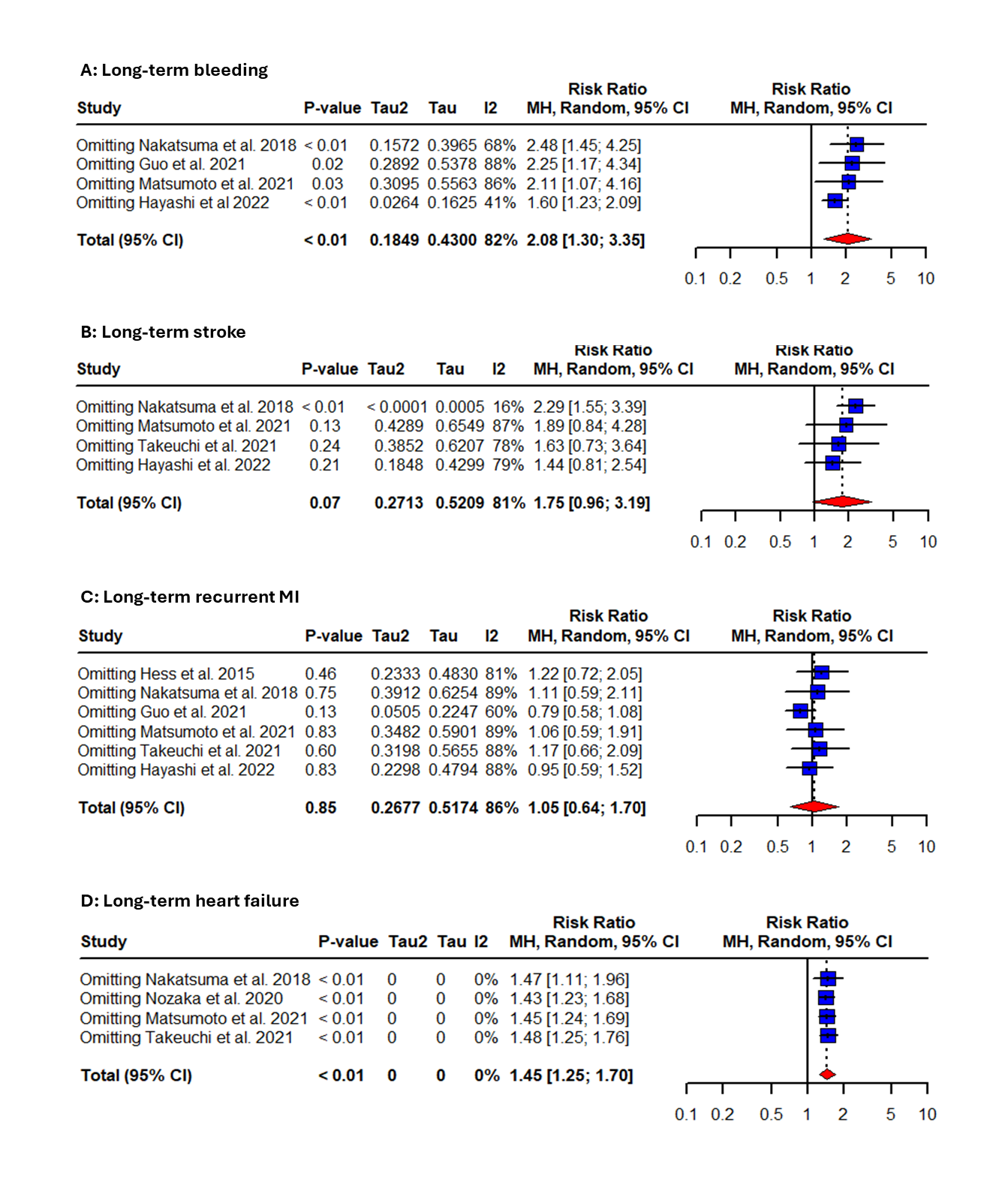


# Supplemental Figure 9: Leave-one-out sensitivity analysis of long-term cardiovascular outcomes by omitting each individual study. (A) long-term bleeding. (B) long-term stroke. (C) long-term recurrent MI. (D) long-term heart failure.


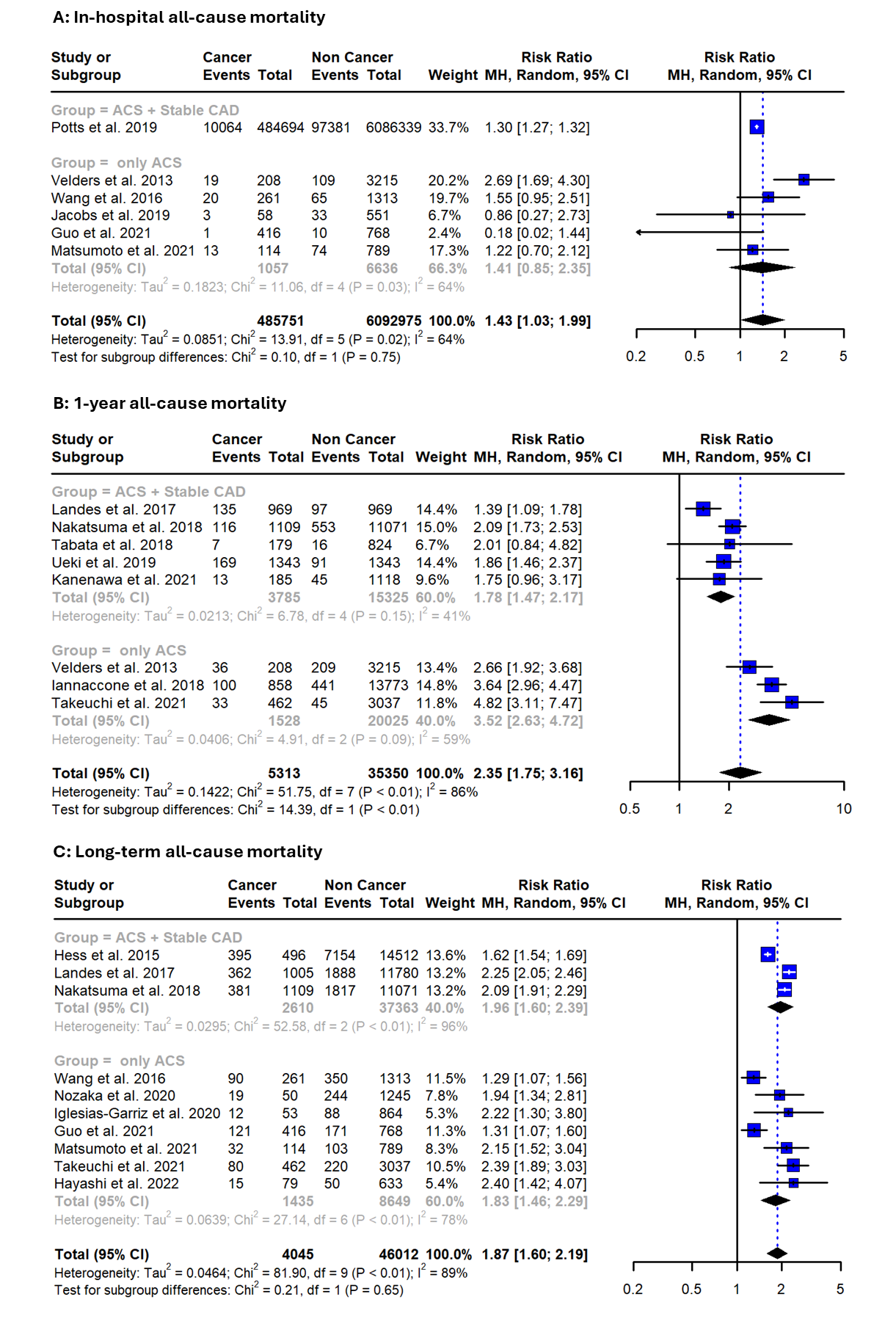


# Supplemental Figure 10: Subgroup analysis of all-cause mortality based on ACS status. (A) in-hospital all-cause mortality. (B) 1-year all-cause mortality. (C) long-term all-cause mortality.


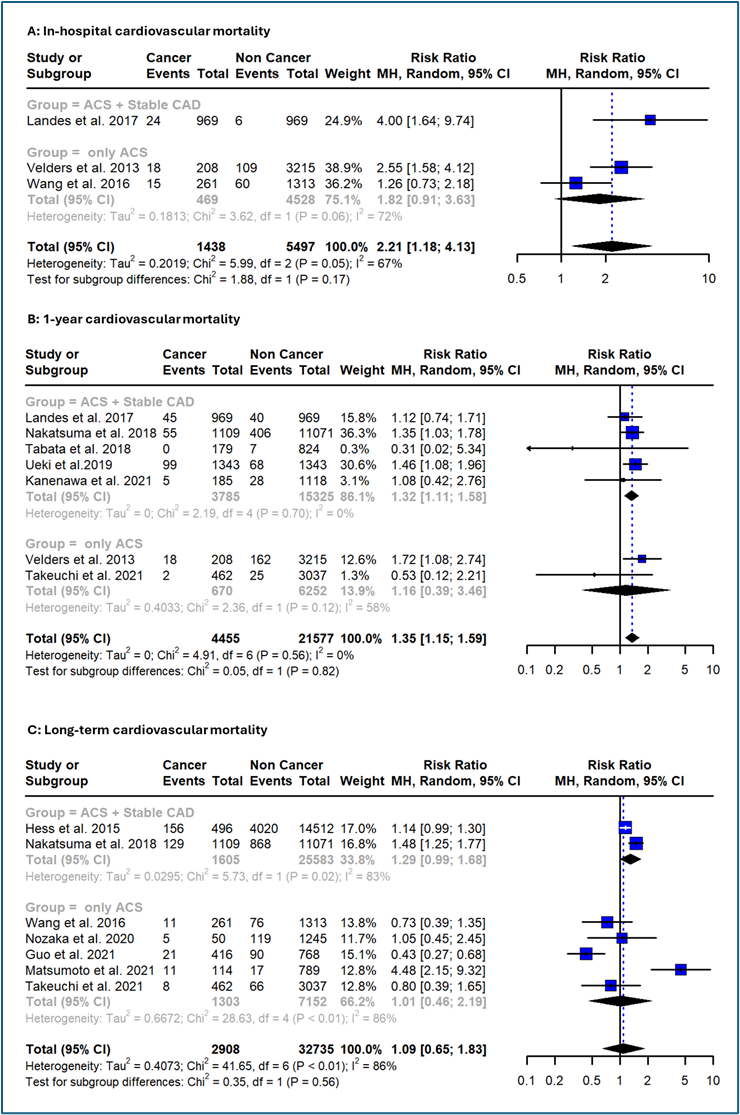


# Supplemental Figure 11: Subgroup analysis of cardiovascular mortality based on ACS status. (A) in-hospital cardiovascular mortality. (B) 1-year cardiovascular mortality. (C) long-term cardiovascular mortality.


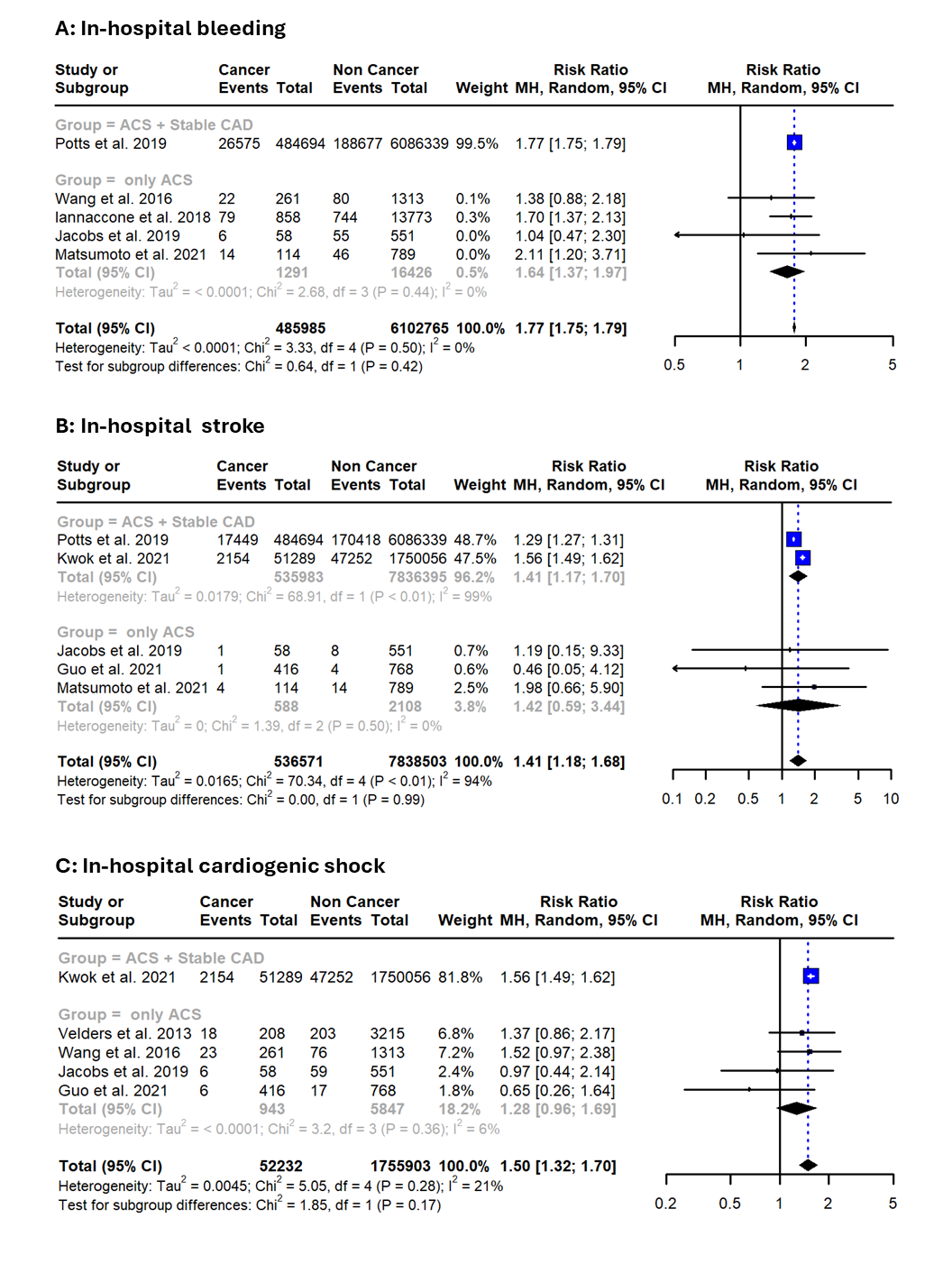


# Supplemental Figure 12: Subgroup analysis of in-hospital cardiovascular outcomes based on ACS status. (A) in-hospital bleeding. (B) in-hospital stroke. (C) in-hospital cardiogenic shock. Studies presenting in-hospital recurrent MI and heart failure events consisted entirely of ACS patients.


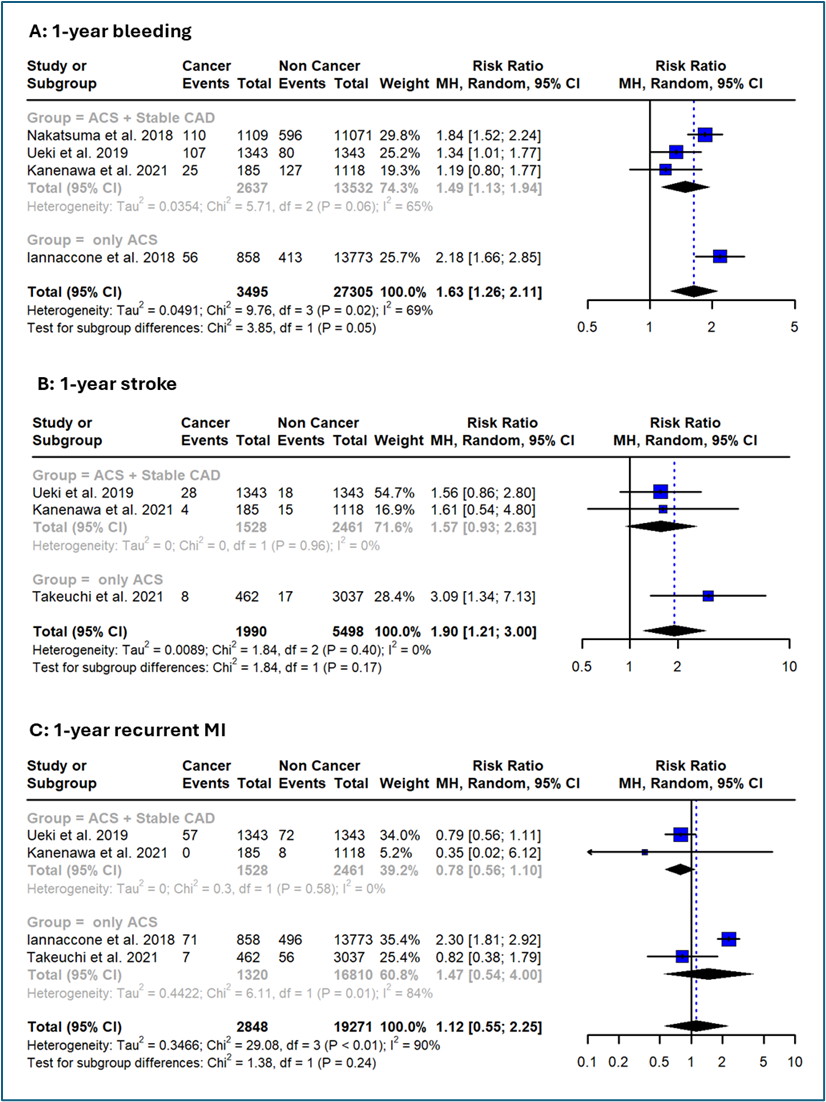


# Supplemental Figure 13: Subgroup analysis of 1-year cardiovascular outcomes based on ACS status. (A) 1-year bleeding. (B) 1-year stroke. (C) 1-year recurrent MI.


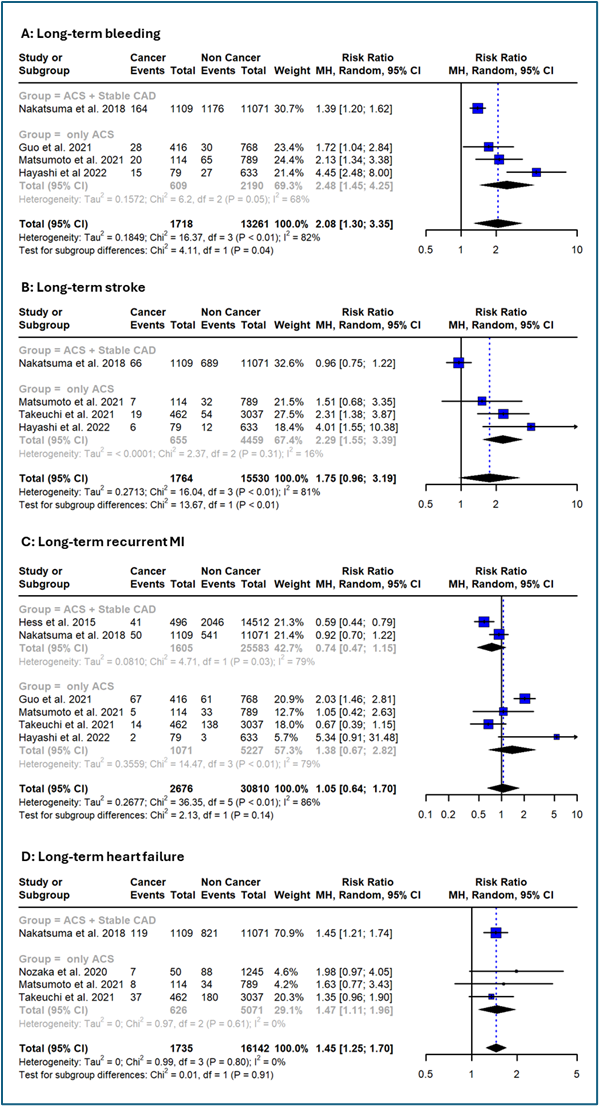


# Supplemental Figure 14: Subgroup analysis of long-term cardiovascular outcomes based on ACS status. (A) long-term bleeding. (B) long-term stroke. (C) long-term recurrent MI. (D) long-term heart failure.


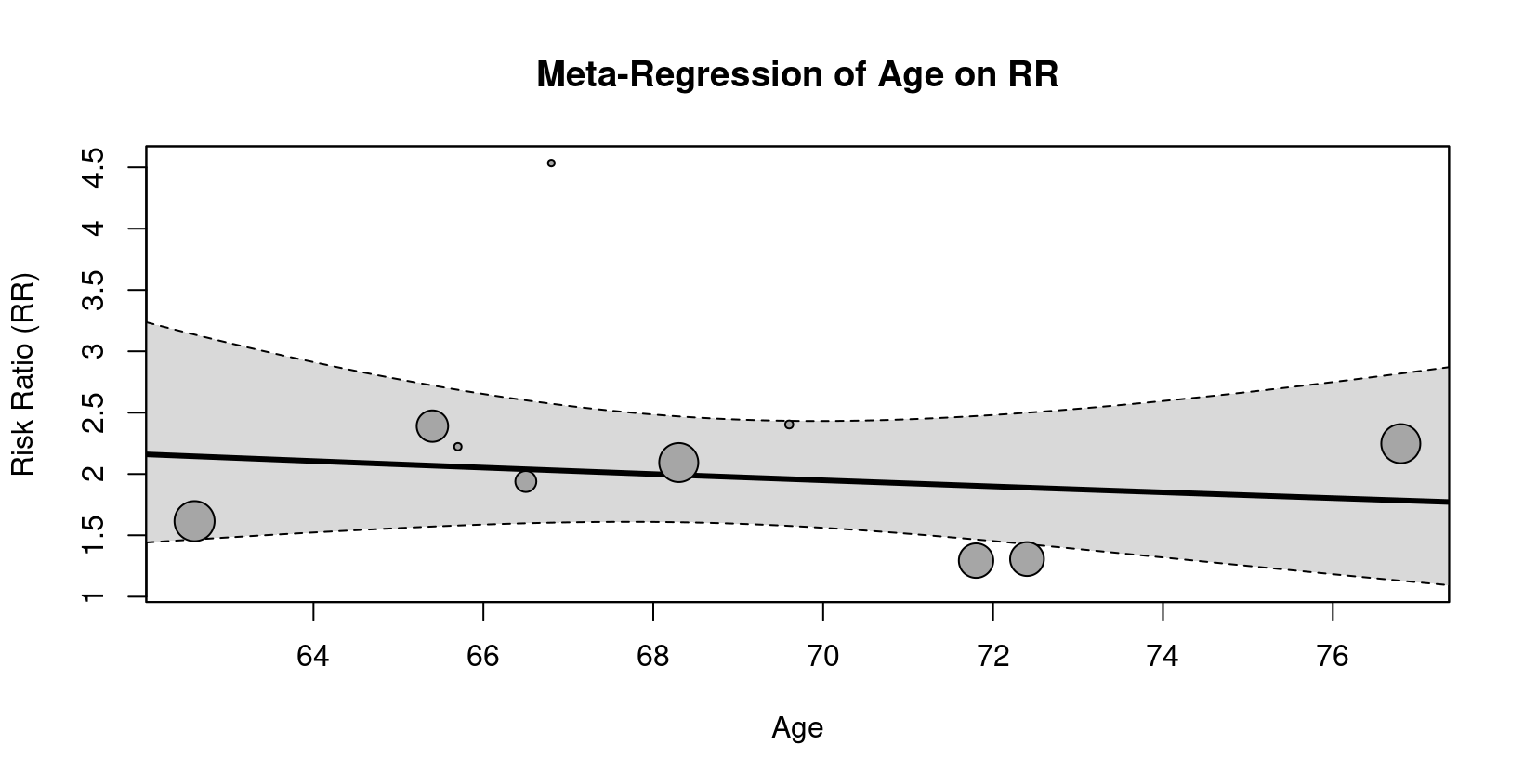


# Supplemental Figure 15: Meta-regression analysis for long-term all-cause mortality with age as covariate.


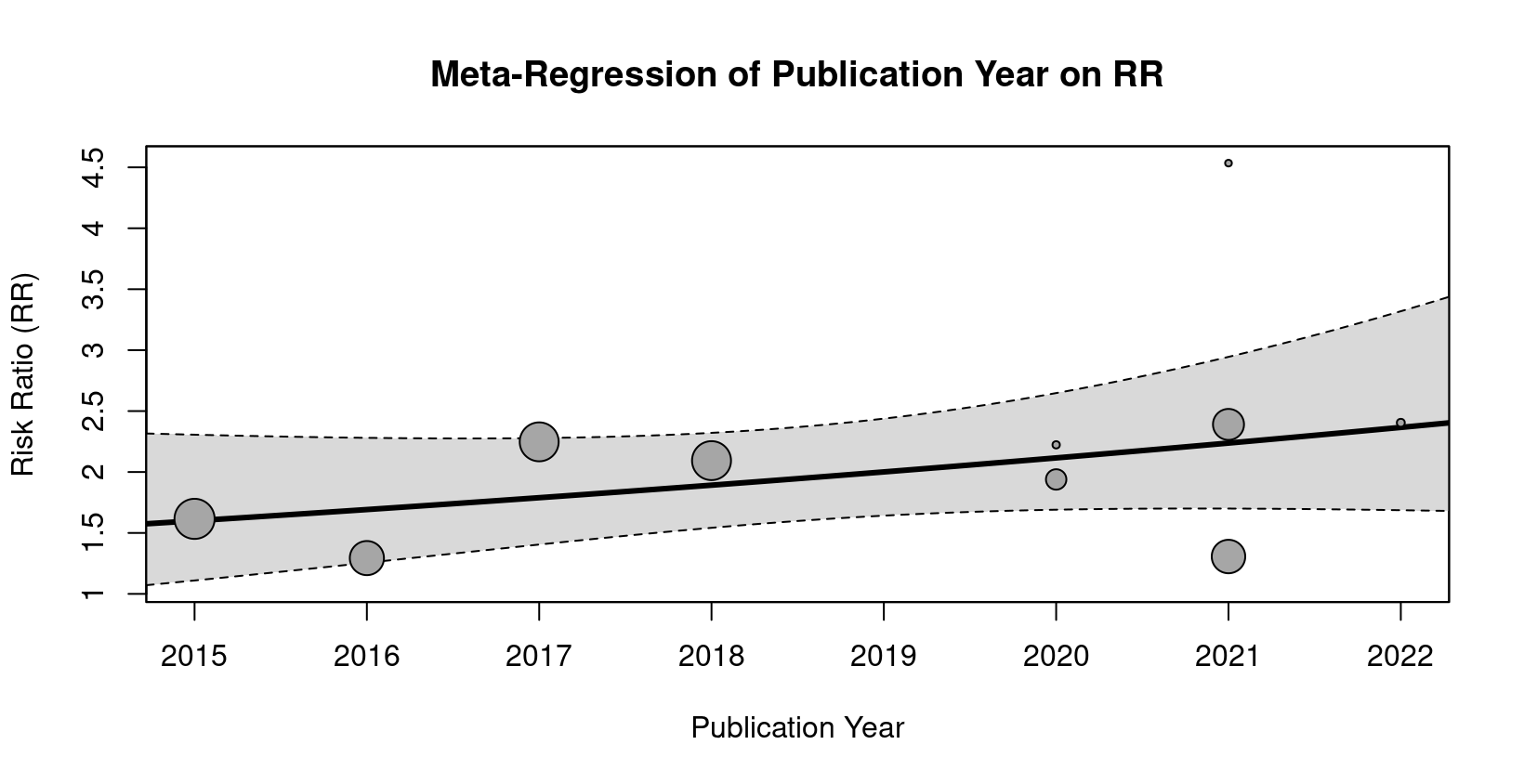


# Supplemental Figure 16: Meta-regression analysis for long-term all-cause mortality with publication year as covariate.
